# A p300/GATA6 axis determines differentiation and Wnt dependency in pancreatic cancer models

**DOI:** 10.1172/JCI156305

**Published:** 2022-06-15

**Authors:** Zheng Zhong, Nathan Harmston, Kris C. Wood, Babita Madan, David M. Virshup

**Affiliations:** 1 Program in Cancer and Stem Cell Biology, Duke–NUS Medical School, Singapore.; 2 Department of Physiology, National University of Singapore, Singapore.; 3 Science Division, Yale–NUS College, Singapore.; 4 Department of Pharmacology and Cancer Biology and; 5 Department of Pediatrics, Duke University, Durham, North Carolina, USA.

**Keywords:** Oncology, Cancer, Drug therapy, Signal transduction

## Abstract

Wnt signaling regulates the balance between stemness and differentiation in multiple tissues and in cancer. *RNF43*-mutant pancreatic cancers are dependent on Wnt production, and pharmacologic blockade of the pathway, e.g., by PORCN inhibitors, leads to tumor differentiation. However, primary resistance to these inhibitors has been observed. To elucidate potential mechanisms, we performed in vivo CRISPR screens in PORCN inhibitor–sensitive *RNF43*-mutant pancreatic cancer xenografts. As expected, genes in the Wnt pathway whose loss conferred drug resistance were identified, including *APC*, *AXIN1*, and *CTNNBIP1*. Unexpectedly, the screen also identified the histone acetyltransferase *EP300* (p300), but not its paralog, *CREBBP* (CBP). We found that *EP300* is silenced due to genetic alterations in all the existing *RNF43*-mutant pancreatic cancer cell lines that are resistant to PORCN inhibitors. Mechanistically, loss of *EP300* directly downregulated *GATA6* expression, thereby silencing the GATA6-regulated differentiation program and leading to a phenotypic transition from the classical subtype to the dedifferentiated basal-like/squamous subtype of pancreatic cancer. *EP300* mutation and loss of *GATA6* function bypassed the antidifferentiation activity of Wnt signaling, rendering these cancer cells resistant to Wnt inhibition.

## Graphical abstract

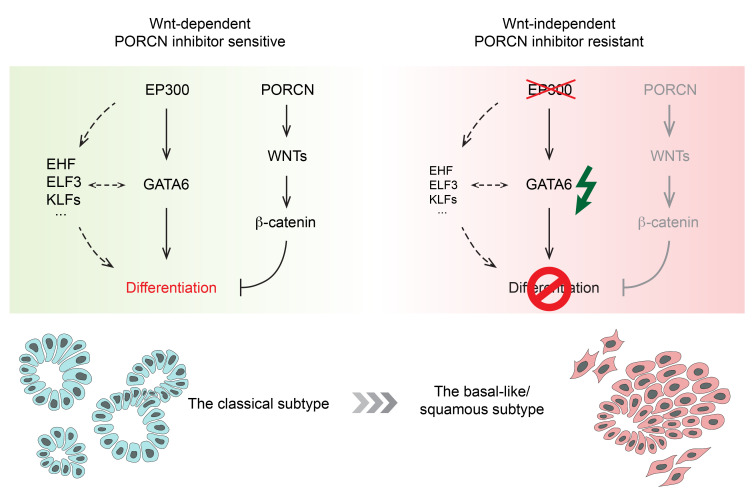

## Introduction

Pancreatic cancer (pancreatic ductal adenocarcinoma [PDAC]) is a lethal disease with a 5-year survival of less than 10%. Most individuals with pancreatic cancer are diagnosed when they have locally advanced or distant metastatic disease, precluding the option of curative surgery (1). The remaining therapeutic modalities, including systemic chemotherapy, provide only limited survival benefits for these patients.

Studies of the precursors of pancreatic cancer have identified *RNF43* mutations in both neoplastic cysts of the pancreas (2, 3) and pancreatic intraepithelial neoplasia (PanIN) (4), and recent advances in genome sequencing have identified loss-of-function *RNF43* mutations in 5%–10% of pancreatic cancers (5–8). *RNF43* encodes a transmembrane E3 ubiquitin ligase that targets cell-surface Wnt receptors, driving their internalization and degradation (9, 10). Inactivation of *RNF43* increases the cell-surface abundance of Wnt receptors such as Frizzleds (FZDs) and LRP5/6. These *RNF43*-mutant pancreatic tumors are therefore hypersensitive to and dependent on ligand-activated Wnt signaling and can be pharmacologically targeted by upstream Wnt pathway inhibition. Small molecule PORCN inhibitors that block the biogenesis of Wnt ligands as well as antibodies that block Wnt function have been shown to inhibit tumor growth in preclinical models of *RNF43*-mutant pancreatic cancers (5, 11), and several have advanced into clinical trials (12).

Wnt signaling plays a critical role in balancing stem cell maintenance with terminal differentiation in various normal tissues (13) as well as in cancers (12). Notably, in multiple preclinical models of Wnt-addicted cancers, Wnt inhibition promoted cancer cell differentiation and loss of cancer stemness rather than cell death (11, 14–18). While differentiation therapy can induce a durable response in selected patient populations, e.g., the hallmark success of treating acute promyelocytic leukemia (APL) by the combination of retinoic acid and arsenic (19), the response to Wnt pathway inhibition in *RNF43*-mutant cancer patients may be heterogeneous. For example, in the recently released phase 1 clinical trial results for WNT974 (LGK974), a first-in-class PORCN inhibitor, while WNT974 successfully controlled disease progression in some cases, several other patients with *RNF43* mutations did not respond or progressed within several weeks of treatment (20). Moreover, in preclinical studies, several pancreatic cancer cell lines with confirmed *RNF43* inactivation were identified that were resistant to PORCN inhibition in cell culture (5). These results suggest that there is intrinsic or acquired resistance to upstream Wnt pathway inhibitors in *RNF43*-mutant pancreatic cancers. Better understanding of these pathways may assist further patient stratification and improve clinical outcomes.

Here, to identify mechanisms that confer resistance to upstream Wnt pathway inhibitors in *RNF43*-mutant pancreatic cancers, we performed in vivo CRISPR screens during PORCN inhibitor therapy in mice bearing *RNF43*-mutant pancreatic tumor xenografts. Validating the screen, several negative regulators of the Wnt/β-catenin signaling cascade were identified. Unexpectedly, we also identified *EP300* (encoding the histone acetyltransferase [HAT] p300) as a regulator of PORCN inhibitor sensitivity. Supporting its importance, we found that *EP300* is inactivated in the preexisting PORCN inhibitor–resistant *RNF43*-mutant pancreatic cancer cell lines. Mutation of *EP300* differs from downstream Wnt pathway mutations that constitutively activate β-catenin signaling because p300 loss confers resistance by mediating Wnt/β-catenin–independent tumor growth. Mechanistically, we found that p300 directly regulates *GATA6* transcription. Loss of p300 downregulates *GATA6* expression, leading to a phenotypic switch from the classical to the basal-like/squamous subtype and intrinsically blocking the GATA6-regulated differentiation program, thereby making the antidifferentiation roles of Wnt signaling dispensable. This identifies a more general role for a p300/GATA6 axis in cancer and suggests that p300 and GATA6 status can be used to stratify *RNF43*-mutant pancreatic cancer patients to maximize the clinical benefits of PORCN inhibitors.

## Results

### Intrinsic resistance to PORCN inhibitors in some RNF43-mutant pancreatic cancers.

**Figure 1:**
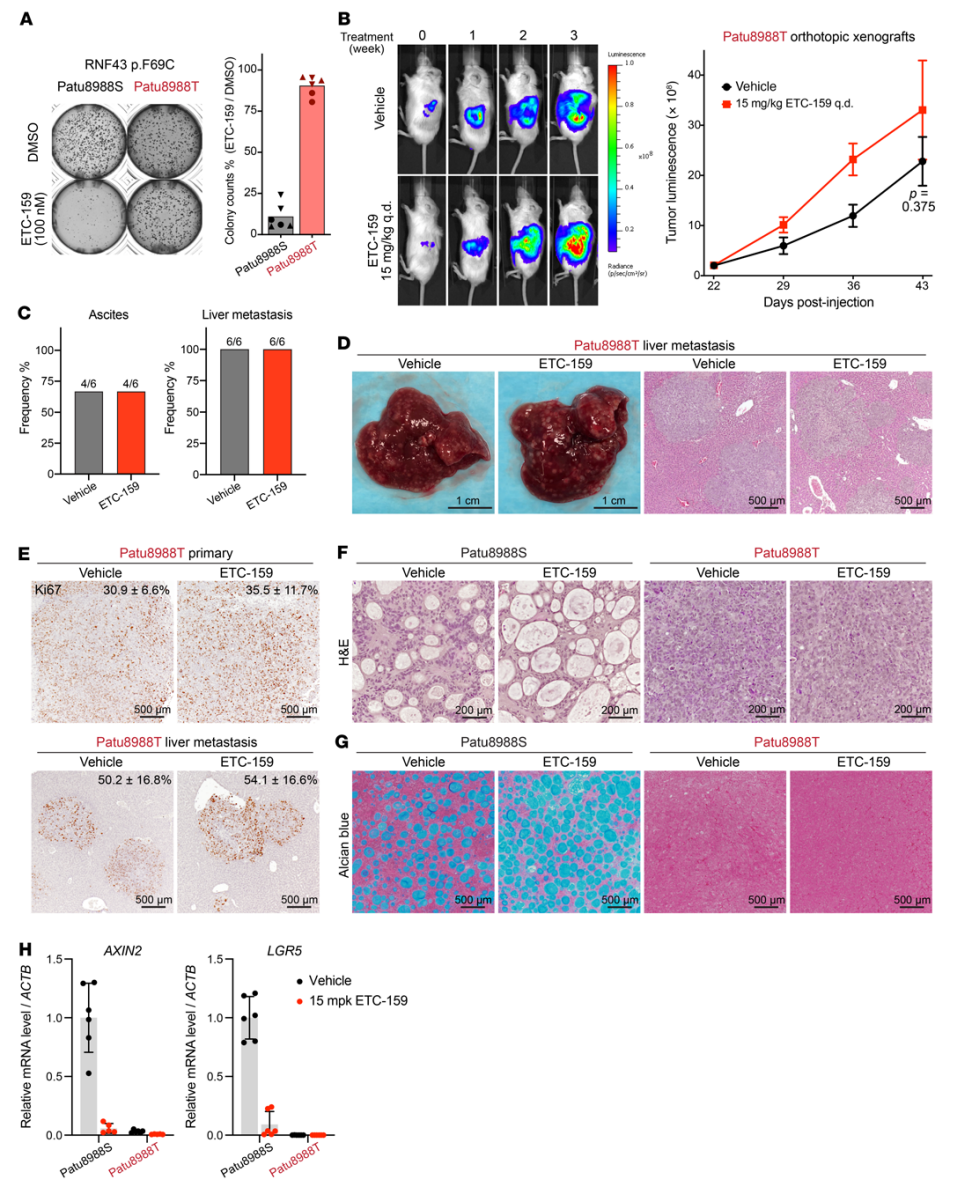
Intrinsic resistance to PORCN inhibitors in some *RNF43*-mutant pancreatic cancers. (**A**) PORCN inhibitor ETC-159 suppressed colony formation of Patu8988S cells, but not Patu8988T cells, in soft agar. Representative images and quantification of colony count from 3 experiments (represented by different symbols) each with biological duplicates are shown. (**B**) Patu8988T orthotopic xenografts are resistant to ETC-159. Patu8988T cells stably expressing luciferase were injected into the pancreas of NSG mice. Three weeks later, tumor-bearing mice were randomized into 2 groups (*n* = 6/group) and treated with vehicle or 15 mg/kg ETC-159 once every day (q.d.). Tumor progression was monitored by measuring luciferase activity weekly. Sequential images from 2 representative mice and luminescence quantification (mean ± SEM) for all mice are shown. *P* value for 2-tailed, unpaired *t* test for tumor luminescence between vehicle and ETC-159 arms at the last time point is shown. (**C** and **D**) ETC-159 did not reduce the development of ascites and liver metastasis in the Patu8988T orthotopic xenograft model. (**C**) Frequency of mice with ascites or liver metastasis at the end of study in **B**. (**D**) Representative gross images of mouse liver with metastasis and H&E staining of the liver sections. (**E**) Ki67 staining of sections of primary Patu8988T orthotopic tumors and liver metastasis. The percentages of Ki67-positive cells (mean ± SD) are shown on top of the images. Six regions from primary tumors of 2 mice per group and 10 liver metastases per group were quantified. (**F** and **G**) H&E staining (**F**) and Alcian blue (pH 2.5) mucin staining (**G**) of sections of Patu8988S and Patu8988T orthotopic tumors. (**H**) Relative mRNA abundance of Wnt target genes *AXIN2* and *LGR5* determined by quantitative reverse-transcription PCR (RT-qPCR) h technical replicates for each sample.

Previous studies have identified a panel of *RNF43*-mutant pancreatic cancer cell lines, several of which have primary resistance to PORCN inhibitors (refs. 5, 21, and Supplemental Figure 1A; supplemental material available online with this article; https://doi.org/10.1172/JCI156305DS1). We examined in depth Patu8988S and Patu8988T cells, sister cell lines that were derived from the same liver metastasis of a pancreatic cancer patient (22). They share the same driver mutations, including *RNF43* p.F69C, *KRAS* p.G12V, and *TP53* pR282W, and these 3 genes underwent loss of heterozygosity (LOH) in both cell lines (23). The RNF43 pF69C mutation has been shown to be dominant negative (5). In a previous study, the PORCN inhibitor WNT974 potently suppressed the growth of Patu8988S cells, but not the Patu8988T cells in 2D culture (5). We confirmed the differential response of these cell lines to Wnt inhibition in soft agar assays using ETC-159, a structurally unrelated PORCN inhibitor also in clinical trials (Figure 1A).

As cancer cells show differential pathway dependencies in vivo versus in vitro (18, 24) and Wnt-target genes are more robustly regulated in vivo versus in vitro (17), we wished to determine whether the differential antitumor efficacy of Wnt inhibition was also seen in mouse xenograft models. However, while both Patu8988S and Patu8988T cells formed small tumors when implanted subcutaneously into the flanks of immunodeficient NOD-scid γ (NSG) mice, they failed to progress. We then implanted these cells orthotopically into the pancreas of NSG mice, better mimicking the tumor microenvironment. There, both Patu8988T and Patu8988S cells formed orthotopic tumors, but only the Patu8988T tumors grew robustly, allowing us to study drug efficacy in this model (Figure 1B). Consistent with the in vitro data, ETC-159 failed to suppress Patu8988T orthotopic tumor growth at a dose that substantially inhibited Wnt signaling (Figure 1B). Patu8988T cells behaved aggressively in the orthotopic model, as the mice developed ascites and liver metastasis, 2 common symptoms of human pancreatic cancer. ETC-159 treatment did not mitigate these events (Figure 1, C and D). Furthermore, Patu8988T cell proliferation in both the primary tumors and the liver metastasis was not suppressed by PORCN inhibition, confirming the in vivo drug resistance of this cell line (Figure 1E).

Previous studies have shown that Wnt deprivation induces differentiation of *RNF43*-mutant pancreatic tumors (5, 11, 17). We therefore analyzed the tissue morphology of Patu8988S and Patu8988T tumors with or without ETC-159 treatment. The tumors derived from these 2 cell lines showed markedly different histology (Figure 1F). Patu8988S tumors formed tubular structure with numerous lumina filled with mucins (Figure 1, E and G), consistent with the exocrine pancreatic origin. ETC-159 treatment led to significant changes in Patu8988S tissue morphology, including increased lumen size and mucin expression and decreased nuclear/cytoplasmic ratio (Figure 1, F and G). This is similar to the tumor differentiation observed in other PORCN inhibitor–sensitive *RNF43*-mutant pancreatic tumors following Wnt inhibition (17). In contrast, Patu8988T tumors were solid tissues with mesenchymal features. ETC-159 treatment did not change their histologic appearance (Figure 1F). Mucin expression was not detected by Alcian blue staining in untreated tumors, and ETC-159 treatment did not induce mucin expression (Figure 1G). In summary, Patu8988S cells formed differentiated tumors and Wnt inhibition further promoted that differentiation, whereas Patu8988T cells formed undifferentiated tumors that were resistant to Wnt inhibition–induced differentiation.

Failure to respond to upstream Wnt pathway inhibition could result from constitutive activation of downstream β-catenin signaling (25). We therefore analyzed Wnt target gene expression in both Patu8988S and Patu8988T tumors with and without PORCN inhibition. ETC-159 potently suppressed *AXIN2* and *LGR5* expression in Patu8988S tumors, but surprisingly, the basal expression levels of these 2 Wnt-target genes were much lower in the Patu8988T tumors (Figure 1H). This suggests that Patu8988T cells are resistant to upstream Wnt inhibition because they have lost dependency on Wnt signaling, the initial driving force of tumorigenesis, through an unknown mechanism.

### In vivo CRISPR screens identified essential genes and tumor suppressors in RNF43-mutant pancreatic cancer.

**Figure 2:**
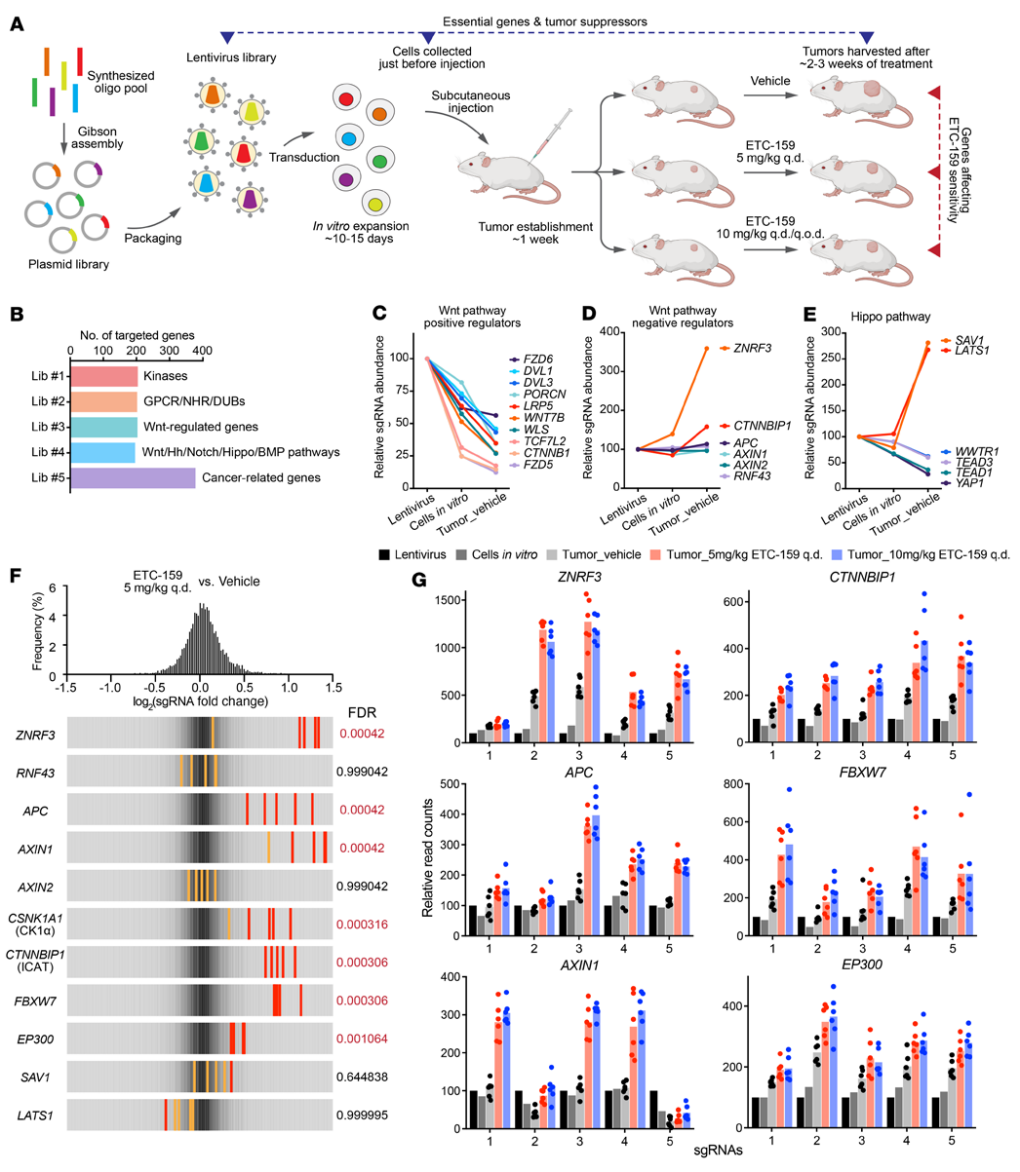
In vivo CRISPR screens identified genes whose loss caused resistance to PORCN inhibition. (**A**) Schematic representation of CRISPR screens in *RNF43*-mutant pancreatic cancer model. Refer to the text for detailed description. (**B**) Custom small-scale sgRNA libraries were designed and constructed for in vivo CRISPR screens. NHR, nuclear hormone receptor; DUB, deubiquitylase; Hh, Hedgehog; Lib, library. Wnt-regulated genes were selected from DE genes upon ETC-159 treatment in HPAF-II tumors (17). (**C**–**E**) Change of the abundance of sgRNAs targeting Wnt and Hippo pathways. Values indicated are the relative median abundance of all the 5 sgRNAs targeting a specific gene. Values are set to 100 for all genes in the lentivirus libraries. (**F** and **G**) In vivo CRISPR screens identified genes, loss of which conferred resistance to PORCN inhibitor ETC-159 in *RNF43*-mutant pancreatic cancer. (**F**) The frequency distribution of each sgRNA abundance ratio (log_2_ fold change) in ETC-159–treated (5 mg/kg q.d.) tumors versus vehicle-treated tumors is shown on the top. Overall frequency distribution of all sgRNAs corresponds to the gray color shown in the bottom panels. For a specific gene, all 5 sgRNAs are shown in the bottom panels. Red line or orange line indicates *P* value calculated by MAGeCK for that specific sgRNA < 0.05 or > 0.05, respectively. Gene level sgRNA enrichment FDRs calculated by MAGeCK are shown next to the panels. (**G**) Individual sgRNA read count change in all the samples for representative hits shown in **F**. For each gene, the 5 sgRNAs are shown. For each sgRNA, its read count in the lentivirus library is normalized to 100. Each dot represents the read count in an individual tumor (*n* = 6).

To identify mechanisms that mediate Wnt-independent growth of *RNF43*-mutant pancreatic cancers, we adapted an in vivo CRISPR screen approach we developed previously (ref. 18 and Figure 2A). For in vivo screens, several factors limit the robust representation of all sgRNAs in a tumor, including the scale of the CRISPR library, the number of cells that can be injected in vivo, the percentage of tumor-initiating cells within the injected cell population, and the intratumor heterogeneity. Our previous study showed that a relatively small scale CRISPR library can be faithfully represented in subcutaneous xenografts of HPAF-II cells, a PORCN inhibitor–sensitive *RNF43*-mutant pancreatic cancer model (18). To identify genes whose loss affected PORCN inhibitor sensitivity, we designed and constructed 4 custom CRISPR lentiviral libraries of approximately 200 genes with 5 sgRNAs per gene (Figure 2B and Supplemental Table 1; please refer to Methods for more details). A fifth library, previously described (18, 26), that targeted 378 cancer-related genes was also included. For each CRISPR library, HPAF-II cells were transduced with the lentivirus library at a low multiplicity of infection (MOI = 0.3) to ensure that the majority of the transduced cells got only 1 sgRNA. The cells were then selected with puromycin, expanded in vitro, and subsequently implanted subcutaneously (10^7^ cells/injection) in NSG mice. After tumor establishment, the tumor-bearing mice were randomized into vehicle or ETC-159–treatment groups. Based on prior experience aiming for tumor growth inhibition of approximately 60% to 70% (18), 5 mg/kg ETC-159 daily by oral gavage was used for all 5 libraries, while 10 mg/kg ETC-159 daily or every other day was used in 2 validation groups (Figure 2A and Supplemental Figure 2A).

To determine the effect of knockout of these approximately 1200 genes on the cell fitness and response to PORCN inhibition, samples from each step in the protocol (Figure 2A) were analyzed by deep sequencing to detect the frequency of all sgRNAs. Due to the small scale of our CRISPR libraries, for each sample, the sgRNA read counts were normalized to the sum of all nontargeting control sgRNAs. Correlation analysis showed variations occurred during the evolution of CRISPR library–carrying cell populations in vitro and in vivo but also showed a high consistency of tumor replicates within each treatment group (Supplemental Figure 3). This allowed a high-resolution statistical analysis of changes in sgRNA abundance.

We first examined what happened to specific sgRNAs in the absence of drug treatment. Depletion or enrichment of specific sgRNAs in the transition from lentivirus library to cells in vitro and subsequently to vehicle-treated tumors identified essential genes and tumor suppressors, respectively (Figure 2A). sgRNAs targeting the positive controls were largely depleted, and the depletion patterns were highly consistent across different libraries, indicating the robustness of the screen (Supplemental Figure 2B). Approximately 15% of the approximately 1200 curated genes in our CRISPR libraries affected HPAF-II cell fitness (Supplemental Table 2), but with diverse dependency patterns in vitro and/or in vivo (Supplemental Figure 2, C and D), highlighting the difference between in vitro and in vivo conditions as well as the advantages of in vivo CRISPR screens. Consistent with the Wnt-dependent nature of this cancer, many components of the Wnt-signaling cascade were essential for cell growth in vitro and in vivo (Figure 2C). The extent of the dependencies was variable, which in some cases might be due to functional redundancy (e.g., *DVL1* and *DVL3*). We also confirmed the previously reported singular dependency of these cancers on *FZD5* expression (Figure 2C, Supplemental Figure 2E, and ref. 11).

Several negative regulators of the Wnt pathway were included in our libraries (Figure 2D). Interestingly, knockout of genes encoding β-catenin destruction complex components, i.e., *APC* and *AXIN1/2*, did not accelerate cell proliferation, indicating that either the function of β-catenin destruction complex had been completely inhibited or β-catenin–mediated transcription machinery had been saturated in *RNF43*-mutant pancreatic tumors. In contrast, knockout of *CTNNBIP1* (also known as inhibitor of β-catenin and TCF4 [ICAT]), whose gene product prevents interaction between β-catenin and TCF factors (27), promoted the in vivo growth of HPAF-II cells, suggesting that the β-catenin/TCF interaction is a rate-limiting step in these cells. Moreover, knockout of *ZNRF3*, the homolog of *RNF43*, markedly promoted HPAF-II cell growth in vitro and in vivo. This could be via enhanced activation of the Wnt/stabilization of proteins (STOP) pathway that stabilizes MYC and cyclins (28) or via stabilization of additional oncogenic targets of ZNRF3 (9). Knockout of YAP/TAZ and their TEAD cofactors inhibited HPAF-II tumor cell proliferation, whereas knockout of their negative regulators *SAV1* and *LATS1* promoted in vivo (but not in vitro) tumor growth (Figure 2E). This highlights the importance of the Hippo/YAP pathway in *RNF43*-mutant pancreatic cancer and is consistent with the observation of YAP dependency in *APC*/*CTNNB1*-mutant colorectal cancers (29).

### In vivo CRISPR screens identified diverse PORCN-inhibitor resistance genes.

To identify genes whose loss affected ETC-159 sensitivity, we next compared the sgRNA distributions in vehicle- and ETC-159–treated tumors. The relative abundance of the bulk of the sgRNAs did not change significantly. However, several sgRNAs were robustly enriched in the ETC-159–treated tumors compared with vehicle-treated tumors, suggesting that knockout of these genes conferred resistance to PORCN inhibition (Figure 2, F and G). These included multiple negative regulators of the Wnt/β-catenin pathway, consistent with the on-target effect of the drug. For example, loss of *ZNRF3* was enriched an additional approximately 2.2-fold in drug-treated compared with vehicle-treated tumors. This may be due to a further increase in the surface abundance of Wnt receptors, making the residual secreted Wnt ligands sufficient to maintain the active Wnt signaling in the presence of ETC-159. As expected, knockout of core β-catenin destruction complex components *APC*, *AXIN1*, and *CSNK1A1* (encoding CK1α) (Figure 2, F and G), causing Wnt ligand–independent stabilization of β-catenin, conferred resistance to PORCN inhibition. Conversely, knockout of *CSNK1A1* in the absence of ETC-159 treatment reduced cell fitness (Supplemental Figure 4B), consistent with the fact that CK1α has multiple substrates besides β-catenin (30, 31). Knockout of *CTNNBIP1* and *FBXW7* also conferred resistance, likely due to downstream activation of β-catenin/TCF transcriptional activity (27) and stabilization of Wnt/STOP targets (28, 32), respectively.

We then asked whether the mutation of these Wnt pathway genes was responsible for PORCN inhibitor resistance in *RNF43*-mutant pancreatic cancers. However, these negative regulators of the Wnt/β-catenin pathway were neither mutated nor silenced in the existing PORCN inhibitor–resistant *RNF43*-mutant pancreatic cancer cell lines (Supplemental Figure 1A) based on genome, exome, and/or transcriptome sequencing results from public databases. AsPC-1 cells do have a heterozygous dominant negative mutation p.R465C, a known hotspot (33), in *FBXW7*, which may explain the lower sensitivity of AsPC-1 cells to the PORCN inhibitor (Supplemental Figure 1B and ref. 18).

It was reported previously that activation of YAP signaling could bypass both β-catenin dependency in colorectal cancer (34) and KRAS dependency in pancreatic cancer (35). While YAP signaling clearly supported the proliferation of *RNF43*-mutant pancreatic cancer (Figure 2E), the sgRNAs targeting YAP suppressors *SAV1* or *LATS1* were not further enriched by ETC-159 treatment (Figure 2F), suggesting that YAP activation could not bypass Wnt dependency in *RNF43*-mutant pancreatic cancer.

### PORCN inhibitor–resistant RNF43-mutant pancreatic cancer cell lines harbor loss-of-function genetic alterations in EP300.

In addition to the negative regulators of the Wnt pathway, we observed significant enrichment of all 5 sgRNAs targeting *EP300* in ETC-159–treated tumors (Figure 2, F and G), suggesting its loss conferred resistance to PORCN inhibition. *EP300* encodes p300 protein, a HAT that acetylates lysines 18 and 27 of histone 3 (H3K18Ac and H3K27Ac) in promoters and enhancers (36–39). Interestingly, while p300 shares high-sequence identity and many substrates with its paralog *CREBBP* (encoding CBP), knockout of *CREBBP* did not cause drug resistance in our CRISPR screen (Supplemental Figure4C). p300 has been described as a tumor suppressor (40, 41), although it is a coactivator for several transcription factors, including the β-catenin/TCF complex (42). Notably, p300 activity has been implicated in resistance to doxorubicin in bladder and breast cancer (43, 44), but no role in bypassing Wnt/β-catenin signaling has been identified.

**Figure 3:**
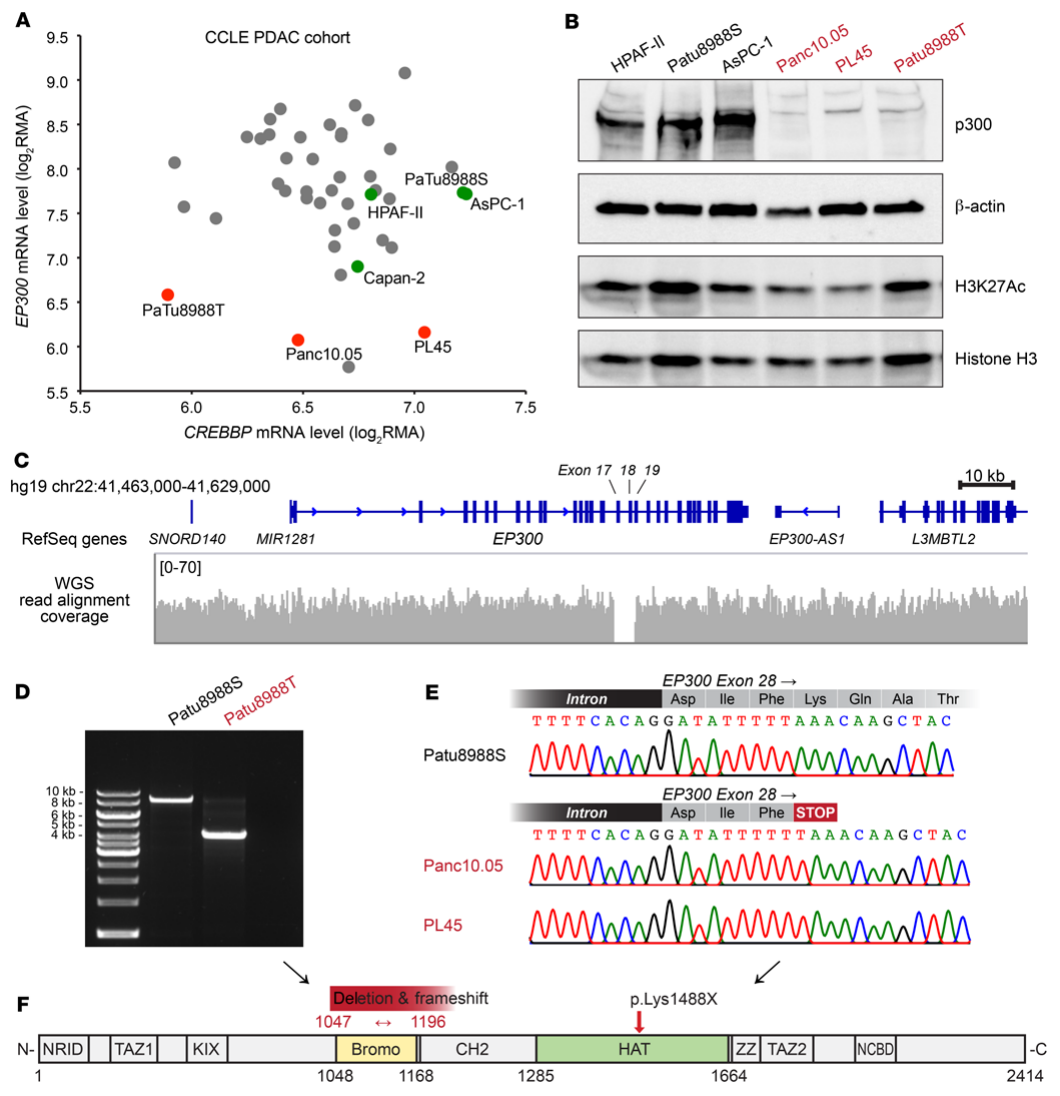
PORCN inhibitor–resistant *RNF43*-mutant pancreatic cancer cell lines harbor loss-of-function genetic alterations in *EP300*. (**A**) mRNA levels of *EP300* and *CREBBP* in a cohort of pancreatic cancer cell lines. The log_2_RMA values of *EP300* and *CREBBP* were extracted from the gene-expression database of CCLE for pancreatic cancer cell lines. Each dot represents an individual cell line. *RNF43*-mutant cell lines are labeled as green dots for PORCN inhibitor sensitive and as red dots for resistant. (**B**) p300 protein and H3K27Ac abundance in a panel of *RNF43*-mutant pancreatic cancer cell lines. (**C**) Visualization of the genomic deletion in the *EP300* locus in Patu8988T cells. Patu8988T WGS data from SRX5466648 (23) were visualized using the Integrative Genomics Viewer (IGV). A zoom-in view of the deleted region is provided in Supplemental Figure 5B. (**D**) The deletion in *EP300* locus is specific to Patu8988T and absent in the sister line Patu8988S. Primers flanking the deleted region (labeled in Supplemental Figure 5B) were used to amplify the genomic DNA from Patu8988T or Patu8988S cells. The expected PCR amplicon from WT is 8362 bp. (**E**) A single nucleotide insertion in *EP300* exon 28 led to frameshift and premature stop codon in Panc10.05 and PL45 cells. Genomic DNA from Panc10.05, PL45, and Patu8988S (as WT control) was PCR amplified. The amplicon was purified and subject to Sanger sequencing. (**F**) The domain architecture of p300 (85). Bromo, bromodomain. The effects of genetic alterations in Patu8988T, Panc10.05, and PL45 on p300 protein are labeled above the architecture illustration.

To investigate whether the resistance to PORCN inhibitors observed in *RNF43*-mutant pancreatic cancer cell lines (Supplemental Figure 1A) was due to loss of p300, we first used the Cancer Cell Line Encyclopedia (CCLE; ref. 23) gene-expression database to analyze the transcript levels of both *EP300* and *CREBBP* in a cohort of pancreatic cancer cell lines (Figure 3A). We found that while the PORCN inhibitor–sensitive *RNF43*-mutant lines had mRNA levels of *EP300* similar to those of the cohort average, the resistant lines were all outliers with *EP300* mRNA levels of approximately 20%–30% of the cohort average (Figure 3A). In contrast, the mRNA level of *CREBBP* could not distinguish these 2 subsets. This observation was replicated in a data set from an independent study (Supplemental Figure 5 and ref. 45). We then assessed endogenous p300 protein abundance in our cell lines by immunoblotting. Strikingly, p300 protein was essentially absent in the PORCN inhibitor–resistant cell lines (Figure 3B). We also analyzed the H3K27Ac abundance as a readout of p300 activity. There was no striking difference in H3K27Ac relative to total histone H3 abundance between the sensitive and resistant cell lines (Figure 3B), suggesting that CBP maintained global H3K27Ac in the absence of p300.

We determined whether there were genetic alterations in *EP300* in the resistant cell lines that might explain the decrease in mRNA and absent protein. Gayther et al. reported *EP300* mutations in approximately 3% of epithelial cancers and cell lines, including a small chromosome deletion in Patu8988T cells (40). This deletion was also seen in a recently released whole genome sequencing (WGS) result of Patu8988T cells from CCLE (Figure 3C, Supplemental Figure 5B, and ref. 23). This genome deletion led to the loss of 3 exons encoding the bromodomain of p300 and a frameshift (Figure 3F). Analyzing our cells with primers surrounding this deleted region (Supplemental Figure 5B), we found that this deletion was specific to Patu8988T and absent in the sister line Patu8988S, which is sensitive to PORCN inhibition (Figure 3D).

To determine whether *EP300* was mutant in other cell lines with primary PORCN-inhibitor resistance, we sequenced the *EP300* gene in Panc10.05 cells. This identified a single nucleotide insertion in exon 28 of *EP300* (Figure 3E), consistent with both LOH and the report in the CCLE database. This insertion led to a premature stop codon that truncated p300 in the middle of the HAT domain that very likely inactivates the enzyme (Figure 3F). For PL45 cells, there is no NGS data available, but they were derived from the same patient as Panc10.05. Indeed, Sanger sequencing showed that PL45 cells harbor an insertion identical to that of Panc10.05 cells (Figure 3E). Additionally, these 3 cell lines have lost the WT allele of *EP300* due to LOH, as revealed by PCR, gDNA sequencing (Figure 3, C–E), and CCLE copy number variation analysis (23). Consistent with the total loss of p300 function, Panc10.05 and Patu8988T cells are the only ones in a panel of pancreatic cancer cell lines that do not tolerate *CREBBP* knockout (Supplemental Figure 5C). Taken together, these findings suggest that *EP300* mutations acquired during tumor evolution cause both nonsense-mediated mRNA decay and the loss of functional p300 protein in the resistant cell lines.

### Loss of p300 mediated Wnt/β-catenin–independent tumor growth in RNF43-mutant pancreatic cancer.

**Figure 4:**
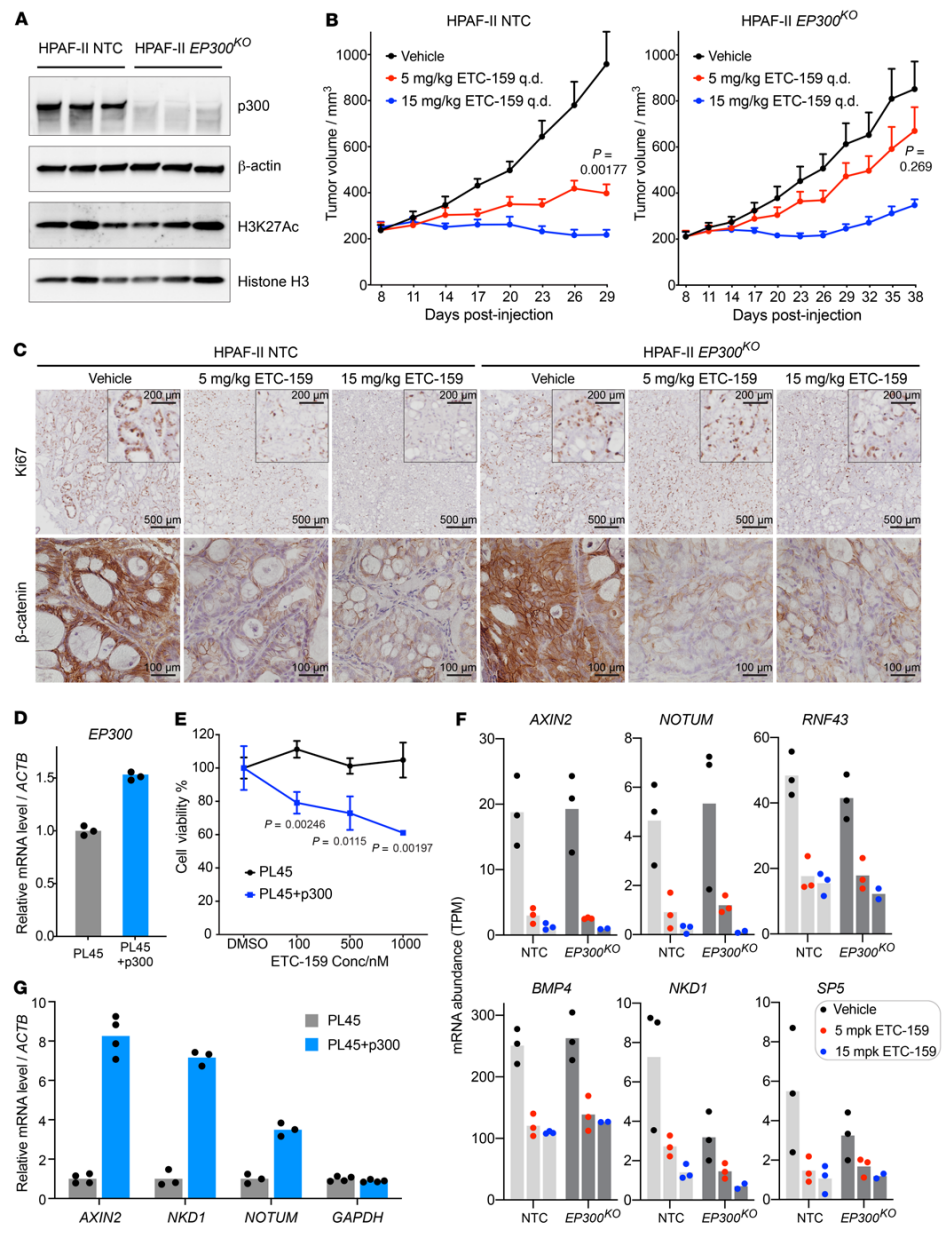
Loss of p300-mediated Wnt/β-catenin-independent tumor growth in *RNF43*-mutant pancreatic cancer. (**A**) p300 protein was undetectable in *EP300*-knockout HPAF-II tumors, while the global H3K27Ac was not affected. Each lane represents an independent tumor. HPAF-II NTC, HPAF-II transduced with a nontargeting control sgRNA. (**B**) *EP300*-knockout HPAF-II tumors were less sensitive to PORCN inhibitor. HPAF-II NTC or *EP300*-knockout cells (Supplemental Figure 6E) were subcutaneously injected into NSG mice. Tumor-bearing mice were treated with vehicle or 5 mg/kg or 15 mg/kg ETC-159 q.d. (*n* = 7–8 tumors/arm) 8 days after injection. Data are represented as mean + SEM. *P* values of 2-tailed, unpaired *t* test between vehicle and 5 mg/kg ETC-159 arms at time of sacrifice are shown. (**C**) Ki67 and β-catenin staining of tumor sections from **B**. (**D** and **E**) Reexpressing WT *EP300* in *RNF43*/*EP300-*mutant PL45 cells rescued Wnt dependency. (**D**) mRNA abundance was assessed by RT-qPCR using primers that detected both endogenous and exogenous *EP300* (*n* = 3 technical replicates). (**E**) Low-density growth of the indicated cells was assessed in DMSO (0.1% v/v) or a concentration gradient of ETC-159 for approximately 2 weeks. Cell viability was measured by crystal violet staining and plotted as mean ± SD (*n* = 3 biological replicates). *P* values of 2-tailed, unpaired *t* test between PL45 and PL45 plus p300 arms are shown. (**F**) Short-term *EP300* knockout did not affect the activity or sensitivity of the Wnt/β-catenin pathway. Orthotopic xenografts of HPAF-II NTC or *EP300*-knockout cells were treated with vehicle or 5 mg/kg or 15 mg/kg ETC-159 q.d. for 3 days. Tumors were harvested 8 hours after the last dose, and gene expression was analyzed by RNA-Seq. Each dot represents an independent tumor. (**G**) Reexpressing WT *EP300* in PL45 cells upregulated Wnt target genes. mRNA abundance was determined by RT-qPCR (*n* = 3–4 technical replicates).

To move from correlation to testing a causal link between p300 loss and resistance to PORCN inhibition, as well as studying the underlying mechanism, we knocked out *EP300* in HPAF-II cells using 2 independent sgRNAs (Supplemental Figure 6A). Sequencing the target sites in both pools of cells demonstrated a high percentage of indels (Supplemental Figure 6, B and C). The pools of knockout cells were significantly less sensitive to 2 structurally unrelated PORCN inhibitors, ETC-159 and C59, in a 2D culture assay (Supplemental Figure 6D), demonstrating that p300 loss confers resistance to the class of PORCN inhibitors rather than to one specific molecule. We subcloned a knockout line with a homozygous frameshift deletion in *EP300* (Supplemental Figure 6E) and complete loss of p300 protein (Figure 4A). Global H3K27Ac abundance was not affected by *EP300* knockout (Figure 4A), consistent with the observation in the primary *EP300*-mutant cell lines (Figure 3B). Moreover, to validate our initial finding from CRISPR screens that *CREBBP* knockout did not confer PORCN inhibitor resistance, we knocked out *CREBBP* in HPAF-II cells with 2 independent sgRNAs (Supplemental Figure 7, A–C) and found that CBP depletion made HPAF-II cells even more sensitive to PORCN inhibitors (Supplemental Figure 7D).

To directly test the role of p300 in the in vivo response to PORCN inhibition, we implanted the control and *EP300*-knockout HPAF-II cells subcutaneously in NSG mice (Figure 4B). While low-dose ETC-159 treatment (5 mg/kg/d) showed a statistically significant approximately 75% inhibition of control tumor growth, it had a minimal and insignificant effect on the growth of *EP300*-knockout tumors. The higher dose (15 mg/kg ETC-159/d) treatment led to tumor shrinkage in both groups, but in the *EP300*-knockout group, all tumors regrew after 2 to 3 weeks. This differential response to PORCN inhibition was further supported by Ki67 staining (Figure 4C). ETC-159 potently inhibited cell proliferation in the WT tumors, whereas in the *EP300*-knockout tumors, cell proliferation remained readily detectable at both dose levels. Thus, we conclude that loss of p300 confers resistance to PORCN inhibition.

We further investigated whether reexpressing WT *EP300* in the existing *RNF43*/*EP300*-mutant pancreatic cancer cell line was able to rescue the Wnt dependency. We generated PL45 cells that stably express WT *EP300* (Figure 4D). While high-dose ETC-159 had no effect on the proliferation of parental PL45 cells, ETC-159 was able to suppress the growth of *EP300*-reexpressing cells in a dose-dependent manner (Figure 4E), supporting the model that *EP300* determines Wnt dependency in *RNF43*-mutant pancreatic cancers. We also attempted to test the in vivo drug sensitivity of the PL45 cell lines, but the xenografts grew extremely slowly in mice, making them unsuitable for testing drug efficacy.

Most of the genes we identified in the screen as conferring resistance to PORCN inhibition are core Wnt pathway genes whose knockout increases β-catenin signaling downstream of PORCN inhibition (Figure 2F). However, p300 loss appears to cause resistance via a different mechanism. In HPAF-II tumors, the knockout of *EP300* did not alter either β-catenin abundance or Wnt target gene expression (Figure 4, C and F). Importantly, Wnt target genes were equally repressed by ETC-159 treatment in control and *EP300*-knockout tumors (Figure 4F). This suggests that the resistance to PORCN inhibition following *EP300* knockout may result from loss of dependency on, rather than alternative activation of, Wnt/β-catenin signaling. Moreover, similarly to what occurred in Patu8988T cells that lost active β-catenin signaling (Figure 1H), Wnt target gene expression levels were low in the PL45 parental cells, but were upregulated after *EP300* reexpression (Figure 4G), consistent with Wnt dependency only in p300 intact cells.

### Downregulation of GATA6 mediated p300 loss-dependent resistance to PORCN inhibition.

**Figure 5:**
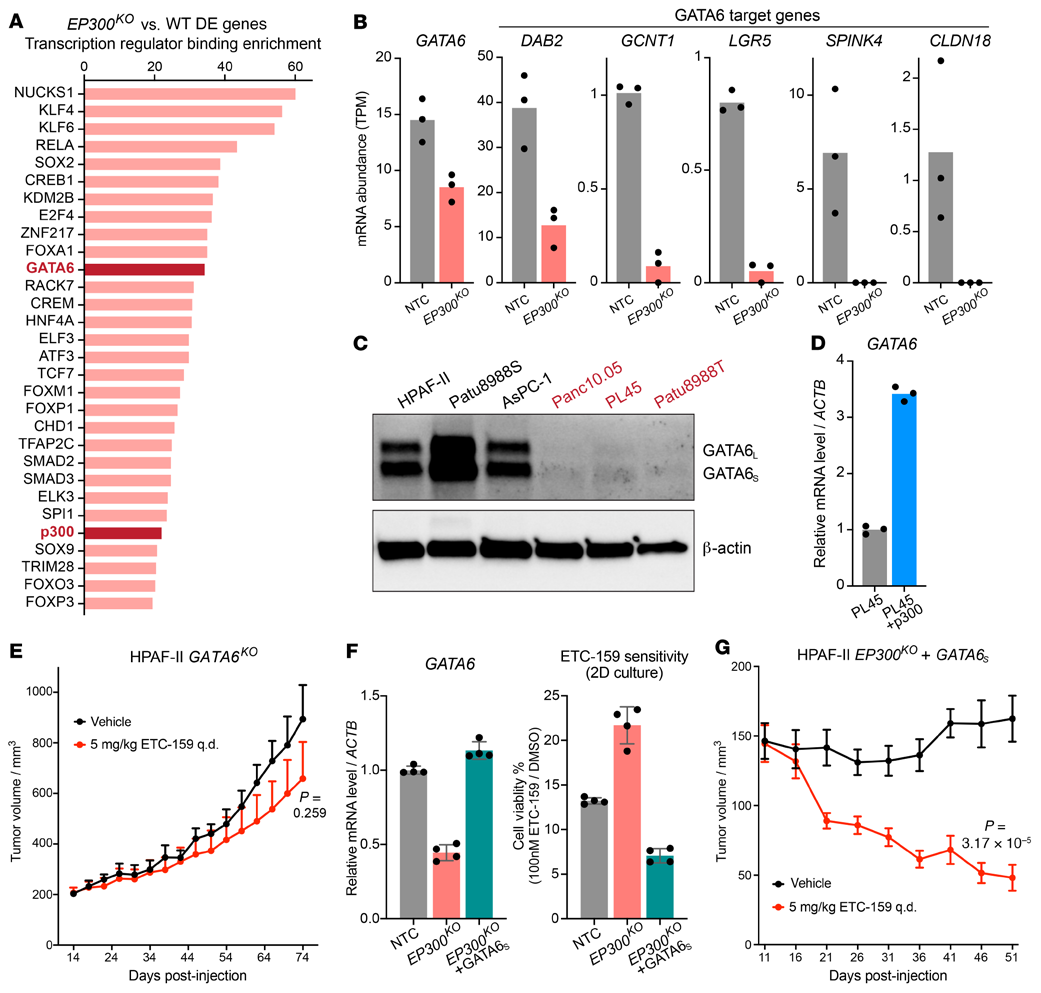
Loss of p300 downregulated GATA6 signaling that determined Wnt dependency. (**A**) Transcription regulator binding enrichment analysis of DE genes. The top 30 enriched transcription regulators are shown. Bars represent the enrichment score. (**B**) *EP300* knockout reduced the expression of *GATA6* and GATA6 target genes in HPAF-II orthotopic tumors. Each dot represents an independent tumor. (**C**) *EP300* mutation correlates with loss of GATA6 protein in *RNF43*-mutant pancreatic cancer cell lines. The PORCN inhibitor–resistant *EP300*-mutant lines are labeled in red. GATA6 has 2 isoforms due to alternative translation start sites. (**D**) Reexpressing WT *EP300* in PL45 cells restored *GATA6* expression. mRNA abundance was determined by RT-qPCR (*n* = 3 technical replicates). (**E**) *GATA6* knockout conferred resistance to PORCN inhibitor in HPAF-II tumors. The experiment was performed as in Figure 4B. HPAF-II cells transduced with sgGATA6 #5 (Supplemental Figure 10B) were used. Treatment was started 14 days after injection. Data are represented as mean + SEM (*n* = 8 tumors/arm). *P* value of 2-tailed, unpaired *t* test for tumor volume at the last time point is shown. (**F** and **G**) Ectopic expression of the short isoform of GATA6 in *EP300*-knockout HPAF-II cells rescued ETC-159 sensitivity in vitro and in vivo. (**F**) *GATA6* mRNA abundance was determined by RT-qPCR (*n* = 4 technical replicates). Cells seeded in 24-well plates at low density were treated with DMSO or 100 nM ETC-159 until the DMSO wells reached confluency. Cell viability was measured by crystal violet staining (*n* = 4 biological replicates). Data are represented as mean ± SD. (**G**) The effect of *GATA6_S_* expression was assessed as in Figure 4B. Treatment was started 11 days after injection. Data are represented as mean ± SEM (*n* = 8 tumors/arm). *P* value of 2-tailed, unpaired *t* test comparing tumor volumes at the last time point is shown.

p300 is a HAT that controls gene expression. To explore which factors/pathways mediate p300 loss-induced resistance to PORCN inhibition, we performed transcriptome analysis of the *EP300* knockout versus WT tumors. *EP300* knockout affected a large portion of the transcriptome in the vehicle-treated tumors (>6000 differentially expressed [DE] genes, FDR < 0.1; ~2600 DE genes, FDR < 0.01) (Supplemental Figure 8 and Supplemental Table 3). While some of these DE genes could be direct targets of p300, the others could be regulated as secondary downstream events. To explore whether there were direct network nodes that regulate these DE genes, we performed a transcription regulator binding enrichment analysis (46, 47). This identified 79 significantly enriched transcription regulators (FDR < 0.05) downstream of *EP300*, with p300 as one of the hits (Figure 5A and Supplemental Table 4).

GATA6 was one of the transcription factors prominently identified in the enrichment analysis (Figure 5A). GATA6 is an essential factor in pancreatic development (48–53), and its expression levels differentiate major pancreatic cancer subtypes (54, 55). *GATA6* expression levels also positively correlate with the dependency on exogenous Wnts in the ex vivo culture of pancreatic tumor organoids (56, 57). Further validating the analysis, the expression of *GATA6* and well-established GATA6 target genes (refs. 58–60 and Supplemental Figure 9) was significantly reduced in the *EP300*-knockout HPAF-II tumors (Figure 5B). Notably, GATA6 protein abundance was also markedly reduced in the *RNF43*/*EP300*-mutant pancreatic cancer cell lines (Figure 5C), and reexpressing p300 in *EP300*-mutant PL45 cells upregulated *GATA6* expression (Figure 5D). Interestingly, in an unrelated breast cancer cell line, MCF7 expression of *GATA6* also decreased upon *EP300* knockdown (Supplemental Figure 10A). However, the paralog CBP does not positively contribute to *GATA6* expression because in the *CREBBP*-knockout HPAF-II cells, the *GATA6* expression levels were slightly elevated (Supplemental Figure 7E). Notably, the mRNA levels of *EP300* were upregulated after *CREBBP* knockout (Supplemental Figure 7E), which is a potential compensation mechanism between p300 and CBP. Therefore, the elevated *GATA6* expression could be a response to the upregulation of p300 expression. Taken together, these data confirm a robust p300/*GATA6* regulatory axis in multiple cell lines.

We next used several approaches to determine whether GATA6 is an important effector of p300 in conferring PORCN inhibitor resistance. *GATA6*-knockout cells were generated (Supplemental Figure 10B) and implanted into NSG mice, who were then treated with a dose of ETC-159 that reproducibly produces approximately 75% tumor growth inhibition in HPAF-II cells (Figure 4B and ref. 18). Importantly, similarly to what was seen in *EP300* knockouts, ETC-159 treatment only minimally slowed down the *GATA6*-knockout tumor growth without reaching statistical significance, confirming that downregulating *GATA6* conferred resistance to PORCN inhibition (Figure 4B and Figure 5E). Conversely, we also determined whether restoring *GATA6* expression in *EP300*-mutant cells restored sensitivity to PORCN inhibitors. We stably expressed the short isoform of GATA6 (*GATA6_S_*) in *EP300*-knockout HPAF-II cells, restoring physiologic levels of *GATA6* mRNA. Critically, this restored their sensitivity to ETC-159 in 2D culture (Figure 5F). While GATA6_S_ overexpression slowed the growth of *EP300*-knockout HPAF-II cells in vivo (Figure 5G), it also restored their sensitivity to PORCN inhibition, as revealed by the xenograft regression upon treatment (Figure 5G). Similarly, overexpressing *GATA6_S_* in *EP300*-mutant PL45 cells markedly slowed down cell proliferation and sensitized PL45 cells to ETC-159 treatment (Supplemental Figure 11). Taken together, these results indicate that downregulation of *GATA6* mediated the PORCN inhibitor resistance caused by p300 loss in *RNF43*-mutant pancreatic cancers.

To further validate the above findings in additional models, we also knocked out *EP300* in CFPAC-1 cells, another *RNF43*-WT pancreatic cancer cell line dependent on Wnt signaling (18). *EP300* knockout also decreased *GATA6* expression in CFPAC-1 cells (Supplemental Figure 10C). Moreover, low-dose ETC-159 slowed down the growth of control CFPAC-1 tumors, while *EP300* knockout and *GATA6* knockout eliminated that effect (Supplemental Figure 10D), again consistent with a role of the p300/*GATA6* axis in Wnt pathway dependency.

We further determined whether the p300/*GATA6* axis is involved in acquired resistance to PORCN inhibition. Mice bearing HPAF-II xenografts were treated daily with 15 mg/kg ETC-159, a treatment that potently suppressed tumor growth for more than 1 month (Supplemental Figure 12A). However, after approximately 1.5 months on continuous daily dosing, almost all of the ETC-159–treated tumors regrew at a rate comparable to that of the vehicle-treated tumors (Supplemental Figure 12A), indicating that drug resistance emerged. Notably, as all tumors of the ETC-159 treatment group regrew at around the same time and at approximately the same rates, this is less likely due to genetic events (e.g., spontaneous mutations in *APC*) that activate downstream Wnt/β-catenin signaling. Indeed, the expression of Wnt target gene *AXIN2* remained suppressed by ETC-159 in these resistant tumors (Supplemental Figure 12B), suggesting that these tumors lost their dependency on Wnt/β-catenin signaling. Interestingly, we observed significant downregulation of mRNA levels of *EP300* and the downstream effector *GATA6* in these resistant tumors (Supplemental Figure 12B), suggesting that these tumors might bypass Wnt dependency by downregulating the p300/*GATA6* axis.

### p300 maintains GATA6 expression via activating its promoter and an intronic enhancer.

**Figure 6:**
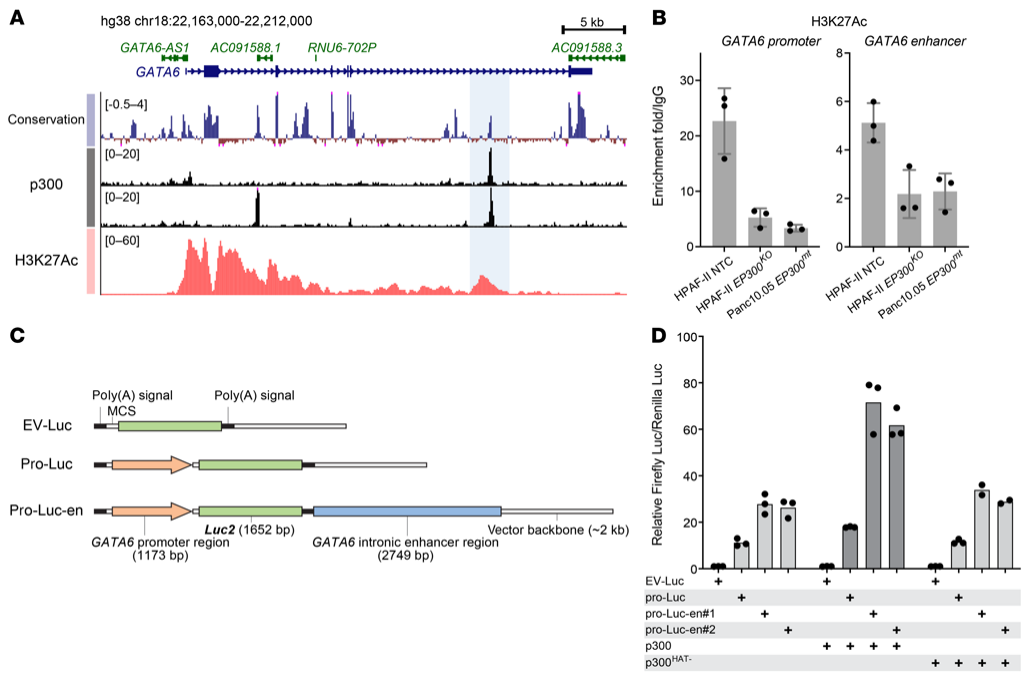
p300 maintains *GATA6* expression via activating its promoter and an intronic enhancer. (**A**) The UCSC conservation track (100 vertebrate species), ENCODE p300 ChIP-seq track (upper: K562 cells, ENCSR000EGE; lower: HepG2 cells, ENCSR000BLW), and H3K27Ac ChIP-Seq track (HPAF-II cells, extracted from GSE64557) in the *GATA6* locus. The potential intronic enhancer region is highlighted. (**B**) *EP300* knockout diminished H3K27Ac in the *GATA6* promoter region and intronic enhancer region. H3K27Ac was assessed by the CUT&RUN assay in HPAF-II NTC, *EP300* knockout, and Panc10.05 cells. Data are represented as mean ± SD (*n* = 3 technical replicates). (**C**) Schematic representation of the luciferase reporter constructs. The *GATA6* promoter region and enhancer region (highlighted in **A**) were cloned into the pGL4.10(*luc2*) vector. MCS, multiple cloning site. *Luc2* encodes a firefly luciferase. (**D**) *GATA6* intronic enhancer sequence and p300 boosted *GATA6* promoter-driven luciferase expression. Dual-luciferase reporter assay using firefly luciferase reporter constructs shown in **C** and a constitutively expressed Renilla luciferase performed in HEK293 cells (*n* = 2-3 biological replicates). WT p300 or a p300 mutant harboring inactivating mutations in its HAT domain (p300^HAT–^) were cotransfected. pro-Luc-en#1/2 are clones with 2 SNVs near a microsatellite site in the enhancer region.

We next investigated how p300 might regulate *GATA6* expression. p300 is a transcriptional coactivator that acetylates histone 3 in promoters and enhancers of active genes to maintain open chromatin and allow Pol II–dependent transcription (38, 39). ENCODE p300 ChIP-Seq data demonstrated that p300 bound to specific domains in both the promoter and an intronic region of *GATA6* (Figure 6A). Notably, the peak in the intronic region was identified in 2 cell lines independently. Supporting a functional role as an enhancer, the sequence of this intronic region is highly conserved in vertebrates (Figure 6A), and its H3K27Ac signal correlates well with the *GATA6* expression levels in both diverse human pancreatic cancer cells and mouse intestine during development (Supplemental Figure 13, A and B). As the readout of p300 activity, we checked publicly available H3K27Ac ChIP-Seq data from HPAF-II cells (Figure 6A). The *GATA6* promoter region is marked with strong H3K27Ac signaling, consistent with active *GATA6* expression. In addition, there is a H3K27Ac peak in the intronic region corresponding to the intronic p300 peak. Using the recently developed CUT&RUN assay, we confirmed H3K27 acetylation in both the *GATA6* promoter and putative enhancer and, importantly, found that *EP300* knockout in HPAF-II cells decreased these signals to that seen in the existing *EP300*-mutant Panc10.05 cells (Figure 6B). Thus, p300 specifically acetylates the *GATA6* promoter and enhancer, and knockout of p300 is not compensated by existing CBP.

To investigate the function of this p300-bound, H3K27Ac-marked intronic region, we cloned its sequence together with the *GATA6* promoter into a luciferase reporter vector (Figure 6C). As expected, adding this intronic sequence significantly enhanced the activity of the *GATA6* promoter in driving luciferase expression (Figure 6D), confirming its role as a potential enhancer. Coexpressing p300 further boosted the transcriptional activity, validating the active involvement of p300 in supporting *GATA6* transcription (Figure 6D). Notably, coexpressing a p300 mutant harboring inactivating mutations in its HAT domain showed no add-on effect (Figure 6D), further highlighting the reliance on p300’s HAT activity in this process.

### Loss of p300/GATA6 axis changes pancreatic cancer subtype and bypasses Wnt dependency.

The above data suggest that loss of p300 or GATA6 bypasses the requirement for β-catenin signaling in Wnt-addicted pancreatic tumors. GATA6 is a well-established master regulator of endoderm development and differentiation as well as pancreas organogenesis and homeostasis (48–53). In pancreatic tumors, GATA6 also promotes cancer cell differentiation and restricts cancer progression in part through maintaining features of the pancreatic lineage (60–63). Based on transcriptomic profiling, pancreatic tumors have been classified into 2 major subtypes: a classical subtype that consists of well/moderately differentiated tumors associated with better disease progression and patient survival; and a basal-like/squamous subtype that consists of poorly differentiated tumors with mesenchymal features and poorer outcome (7, 64–67). Importantly, *GATA6* is a robust marker that is highly expressed in the classical subtype and is almost absent in the basal-like subtype (54, 55).

**Figure 7:**
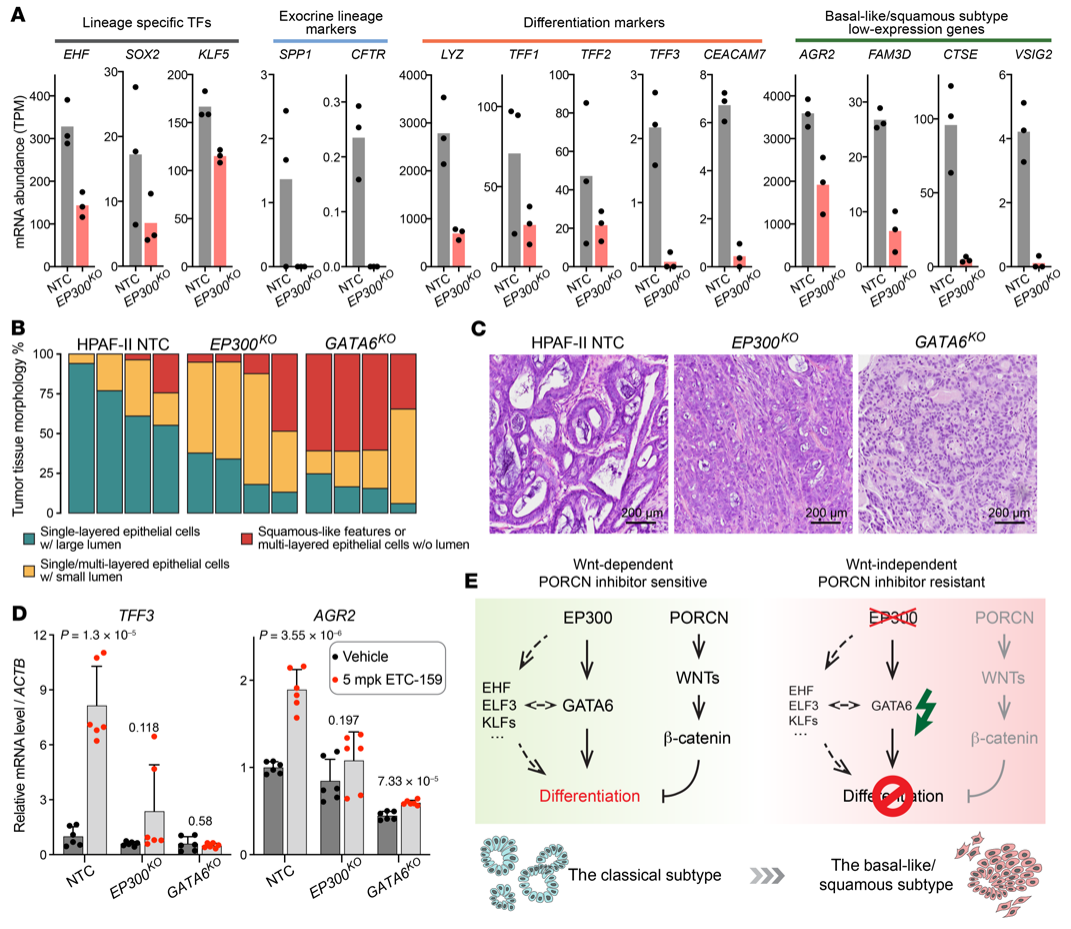
Downregulation of p300/GATA6 led to pancreatic cancer subtype transition and Wnt independence. (**A**) mRNA expression levels of pancreatic lineage, differentiation-related, epithelial, and classical subtype genes in orthotopic xenografts of HPAF-II NTC cells or *EP300*-knockout cells analyzed by RNA-Seq. Data are represented as mean and individual data points. Each dot represents an independent tumor. (**B** and **C**) *EP300* or *GATA6* knockout changed tissue morphology in HPAF-II tumors. Vehicle-treated tumors from Figure 4B and Figure 5E were subject to H&E staining. (**B**) The entire sections were scanned and the areas of regions with the defined tissue morphology were quantified. Tumors from 4 mice per group were analyzed. (**C**) Representative images of H&E staining results are shown. (**D**) *EP300* or *GATA6* knockout prevented upregulation of differentiation genes. *TFF3* and *AGR2* mRNA abundance was measured in control and ETC-159–treated subcutaneous HPAF-II xenografts (Figure 4B and Figure 5E). Three independent tumors per group were analyzed by RT-qPCR with technical replicates for each sample. *P* values of 2-tailed, unpaired *t* test between vehicle and ETC-159 treated samples are shown. (**E**) Schematic model of the p300/GATA6 differentiation axis whose deficiency bypasses Wnt dependency. Refer to the text for detailed description.

We determined whether p300 loss and hence GATA6 suppression led to dedifferentiation in pancreatic cancer. Indeed, the expression levels of multiple differentiation-related lineage and epithelial genes trended downwards in *EP300*-knockout HPAF-II tumors (Figure 7A). These downregulated genes included several whose expression is low in the basal-like/squamous subtype, indicating a transition from the classical to the basal-like subtype after *EP300* knockout. Consistent with this, the expression of the classical subtype genes was low in the panel of *RNF43*/*EP300*-mutant cell lines compared with the *RNF43*-mutant cells with intact p300 (Supplemental Figure 14A).

To further assess the subtype transition, we analyzed the tumor tissue histology (Figure 7, B and C). WT HPAF-II tumors showed tubular structures in most of the regions, reflecting the nature of well-differentiated low-grade pancreatic tumors. In contrast, the number of lumina in *EP300*-knockout tumors was clearly reduced and these tumor cells tended to form solid multilayers with vague epithelial features (Figure 7, B and C). The histologic transition was more pronounced in *GATA6*-knockout tumors where well-organized epithelial tubular structures were rare and mesenchymal features were observed (Figure 7, B and C). A similar phenotypic transition was observed in CFPAC-1 cells after *EP300* or *GATA6* knockout both in vitro and in vivo (Supplemental Figure 10, E and F). Notably, the morphological differences in these isogenic tumors (Figure 7C and Supplemental Figure 10F) paralleled those observed between the Patu8988S and Patu8988T tumors (Figure 1F). The PL45 (*EP300* mutant) xenograft tumors similarly had squamous/mesenchymal histology (Supplemental Figure 14B). Taken together, these results indicate that loss of p300 induced a phenotypic transition (dedifferentiation) of pancreatic cancer, mediated at least in part by the downregulation of *GATA6*.

Consistent with the antidifferentiation role of Wnt signaling, in WT HPAF-II tumors, PORCN inhibition promoted the expression of *TFF3* and *AGR2* (Figure 7D), 2 well-established differentiation markers. Importantly, *EP300* knockout significantly mitigated this effect (Figure 7D), suggesting a blockade of the differentiation program. Moreover, *GATA6* knockout phenocopied what we observed in the *EP300*-knockout tumors with and without ETC-159 (Figure 7D), consistent with the p300/GATA6 axis. Overall, in the presence of WT p300, *GATA6* expression is maintained and the GATA6-induced differentiation (probably coordinated by additional lineage-specific factors; ref. 68) is antagonized by and requires Wnt signaling. In the absence of p300, *GATA6* expression decreases, leading to dedifferentiation and the loss of dependency on Wnt signaling for tumor maintenance (Figure 7E).

Notably, while silencing of the p300/GATA6 axis led to downregulation of the classical subtype gene signature (Figure 7A and Supplemental Figure 14A) and tumor morphological changes showing squamous features (Figure 1F, Figure 7, B and C, and Supplemental Figure 14B), we did not observe consistent changes in the basal-like subtype signature genes after *EP300* knockout in HPAF-II tumors by our RNA-Seq. This pattern was also seen when we compared the existing *RNF43*/*EP300*-mutant pancreatic cancer cell lines with the *EP300*-WT cell lines (Supplemental Figure 15A). Several other factors besides GATA6 have been implicated in regulating subtype switches. Thus, depletion of *KDM6A* (69), activation of GLI2 or YAP1 signaling (70, 71), and overexpression of *TP63* (72) have been reported to contribute to the basal-like subtype in pancreatic cancer. However, these factors are not related to the Wnt-independency phenotype in *RNF43*-mutant pancreatic cancer, since sgRNAs targeting *KDM6A* or negative regulators of GLI2 or YAP1, i.e., *PTCH1*, *LATS1*, and *SAV1*, were not enriched in ETC-159 versus vehicle-treated tumors (Figure 2F and Supplemental Figure 15B) in our initial CRISPR screens. Furthermore, the expression levels of these factors and their regulators are similar between the drug-sensitive and the drug-resistant cell lines (Supplemental Figure 15C). The lack of upregulation of the basal subtype genes in *EP300*-mutant cancers suggests this represents a distinct subclass of the basal-like/squamous subtype.

### Wnt/β-catenin signaling is low in basal-like/squamous subtype pancreatic tumors.

While genetic alterations in *EP300* occur in less than 2% of treatment-naive pancreatic tumors, cooccurrence of *EP300* and *RNF43* mutations has been observed in resected pancreatic tumors (6–8). For example, in a library of patient-derived pancreatic tumor organoids (56), one *RNF43*-mutant organoid line, PC43, grew independently of Wnt signaling. Inspection of the whole-exome sequencing and gene-expression data from this study demonstrates that indeed PC43 harbors both a frameshift mutation and a missense mutation in *EP300* as well as low expression of *GATA6*. These observations in clinical samples are consistent with a key role of the p300/GATA6 axis in determining Wnt dependency.

**Figure 8:**
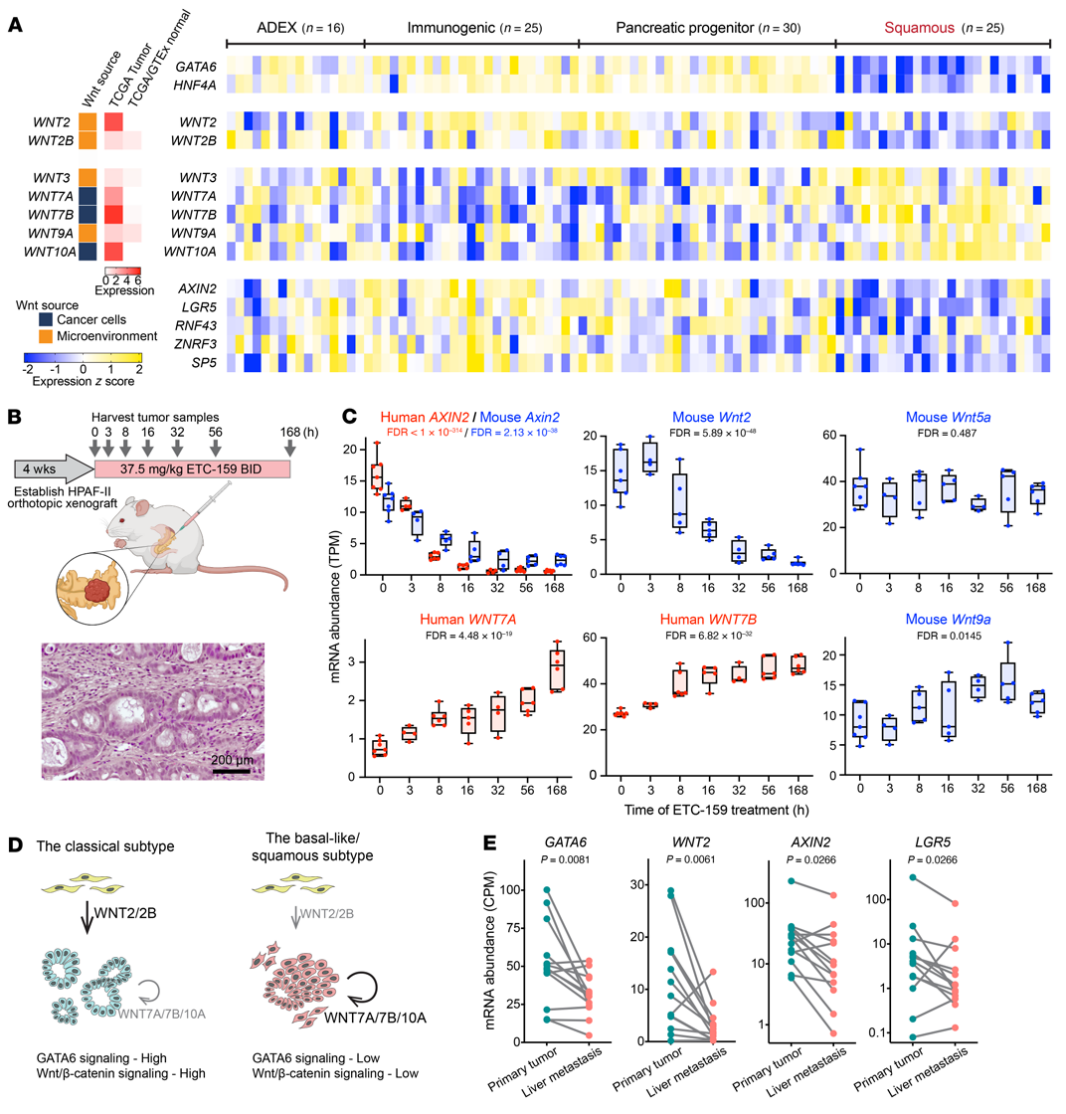
Wnt/β-catenin signaling is low in basal-like/squamous subtype pancreatic tumors and metastases. (**A**) Relative expression levels of lineage factors (*GATA6* and *HNF4A*), detectable Wnt ligands, and selected Wnt target genes (*AXIN2*, *LGR5*, *RNF43*, *ZNRF3*, and *SP5*) in a cohort of treatment-naive primary pancreatic tumors, reanalyzing data from ref. 7, and normalized as *z* score. Tumors were classified into 4 subtypes according to the original study. Abnormally differentiated endocrine exocrine (ADEX), immunogenic, and pancreatic progenitor subtypes largely correspond to the classical subtype. Wnt ligands were classified as cancer cell derived or tumor microenvironment derived, based on single-cell RNA-Seq results of pancreatic tumors (Supplemental Figure 16 and ref. 73). The median expression levels of each Wnt in pancreatic tumors of the TCGA cohort and normal pancreatic tissues from TCGA and GTEx databases were extracted through the GEPIA website. (**B**) Schematic representation of the transcriptomic study in HPAF-II orthotopic xenografts in response to PORCN inhibition (17). H&E staining of a HPAF-II orthotopic tumor shows a large amount of mouse stromal cells surrounding the glandular structures of HPAF-II epithelial cancer cells. (**C**) Diverse responses to Wnt pathway inhibition of Wnt ligand expression. The mRNA abundances of human genes expressed by HPAF-II cells or mouse genes expressed by stromal cells in orthotopic tumors following ETC-159 treatment are shown. Data are represented as box plots. Each dot represents an independent tumor (*n* = 4–7/time point). FDRs computed using the Benjamini-Hochberg procedure are shown. (**D**) Schematic model of Wnt expression patterns and corresponding GATA6 and Wnt/β-catenin signaling in pancreatic tumors. (**E**) GATA6 signaling and Wnt/β-catenin signaling are low in liver metastases of pancreatic cancer. mRNA abundances of *GATA6*, *WNT2*, and Wnt target genes were extracted from GSE151580 (76) for pancreatic primary tumors and matched liver metastases from the same patients (*n* = 13). *P* values of Wilcoxon’s matched-pairs signed rank test are shown.

We speculated that there is a general Wnt dependency in the classical subtype of pancreatic cancer, a subset of which are hyper–Wnt addicted due to *RNF43* mutations. Consistent with this, the Sato and Clevers labs reported that the *GATA6*-high classical subtype tumor cells from *RNF43*-WT pancreatic cancers rely on Wnts to grow as organoids ex vivo. Conversely, *GATA6*-low basal-like/squamous subtype tumor organoids grow ex vivo independently of Wnt signaling (56, 57). This raises the question of where the Wnts in the cancers come from, the cancer cells or the tumor microenvironment. We classified the source of the Wnts using publicly available single-cell RNA-Seq data from pancreatic cancers (Figure 8A, Supplemental Figure 16, and ref. 73). We examined the expression of these Wnts and Wnt/β-catenin target genes in treatment-naive pancreatic cancers where transcriptomic data are available (7). These cancers fall into the 2 main subtypes as before, with clear differential expression of the lineage factors *GATA6* and *HNF4A* (Figure 8A). Interestingly, the detectable Wnt ligands showed 3 distinct expression patterns (Figure 8A and Supplemental Figure 17). *WNT2* and *WNT2B* produced by the tumor microenvironment had high expression in the classical subtype tumors, but low expression in the squamous subtype. Conversely, the cancer cell–derived *WNT7A*, *WNT7B*, and *WNT10A* and stromal *WNT3* and *WNT9A* were highly expressed in the squamous subtype. These 2 groups of Wnts, of stromal and cancer cell origin, were found by Seino et al. to be able to support ex vivo pancreatic organoid growth (56). A third group of Wnts, including *WNT4*, *WNT5A*, *WNT5B*, and *WNT11*, had similar expression levels in the 2 subtypes (Supplemental Figure 17), but did not have the ability to support organoid growth (56).

We asked whether the expression of the different Wnts correlated with active Wnt/β-catenin signaling in the cancers. Importantly, while both subtypes of tumors had their own preferentially expressed Wnt ligands, only the classical subtype tumors with *WNT2* and *WNT2B* expression appeared to have active β-catenin–driven transcription events, as evidenced by the high expression of well-established Wnt/β-catenin target genes, such as *AXIN2* and *LGR5* (Figure 8A and Supplemental Figure 18). Conversely, the cancers with high cancer cell–derived Wnts in the squamous subtype had low expression of Wnt target genes. These data are consistent with functional Wnt/β-catenin signaling being present only in the classical subtype pancreatic tumors.

It was curious that *GATA6*-low squamous tumors had high levels of a subset of Wnts (*WNT3*, *WNT7A*, *WNT7B*, *WNT9A*, and *WNT10A*), yet these tumors were Wnt independent and resistant to PORCN inhibition. We therefore carefully examined the dynamics of expression of these Wnts. Previously, we reported the transcriptomic dynamics of HPAF-II orthotopic xenografts in response to Wnt inhibition (17). Because the stroma in this model came from the mouse pancreas, we could assess by the sequence of the transcripts which Wnts were made in the murine microenvironment and which in the human cancer cells (Figure 8B). Interestingly, we found both positive and negative feedback loops. Inhibiting Wnt signaling downregulated the transcription of stromal *Wnt2*, but upregulated the transcription of stromal *Wnt9a* and epithelial *WNT7A* and *WNT7B* (Figure 8C). This is consistent with the trend observed in the clinical samples (Figure 8A) showing that Wnt/β-catenin–high classical subtype tumors have higher expression of stromal *WNT2*, whereas Wnt/β-catenin–low squamous subtype tumors have higher expression of epithelial *WNT7A* and *WNT7B* and stromal *WNT9A* (Figure 8D). Stromal *Wnt5a*, the Wnt that is highly expressed in both subtypes with no difference, did not respond to ETC-159 (Figure 8C). While the underlying mechanisms remain to be studied, it suggests that the high expression of certain Wnts such as *WNT7A* and *WNT7B* in the squamous subtype tumors is a response to the low Wnt/β-catenin signaling status and that these Wnts, unlike *WNT2* and *WNT2B*, are not sufficient to drive β-catenin signaling in the tumors (Figure 8D).

Our data suggest a p300/GATA6 differentiation axis, where loss of this axis leads to tumor dedifferentiation. Cancer cell dedifferentiation is closely associated with metastasis (74, 75). Indeed, in our study, the established *EP300*-mutant *GATA6*-low cell lines Patu8988T and PL45 formed numerous metastases in vivo (Figure 1, C and D and Supplemental Figure 14B), consistent with the reported antimetastatic role of *GATA6* in preclinical models of pancreatic cancer and the reduced *GATA6* expression level in metastases of pancreatic cancer patients (62, 63). Similarly, when we analyzed a recently released data set of pancreatic primary cancers and matched liver metastases (76), we saw downregulation of both *GATA6* expression and Wnt/β-catenin signaling in the liver metastases (Figure 8E). These further validate the loss of GATA6 signaling and Wnt/β-catenin signaling during the progression of human pancreatic cancers.

## Discussion

Activation of the Wnt/β-catenin pathway by diverse mechanisms supports the progression of many cancers. Increasingly effective approaches to targeting this pathway are being developed. Here, we examined why some *RNF43*-mutant pancreatic tumors are insensitive to Wnt pathway inhibition, using a potent drug currently in clinical trials. In vivo CRISPR screens identified well-known downstream regulators of Wnt/β-catenin signaling as potential causes of drug resistance, but in addition, identified a p300/GATA6 differentiation axis. Loss of p300 led to loss of GATA6 expression, dedifferentiation of the cancers, and a molecular subtype switch. These dedifferentiated tumors no longer relied on Wnt/β-catenin signaling to maintain stemness and prevent differentiation. These findings are consistent with the resistance to PORCN inhibitors of *RNF43*/*EP300*-mutant pancreatic cancer cells that show squamous features.

Of note, a previous study found that in ex vivo culture of pancreatic tumor organoids, downregulation of *GATA6* promoted the expression of cancer cell–derived Wnt ligands, e.g., *WNT7B*, making some of these organoids “exogenous Wnt”-independent (56). However, this “enhanced autocrine” model could not explain the complete Wnt/β-catenin independence they observed in a panel of *GATA6*-low organoids (termed “WRi” in the study; ref. 56). Their findings of PORCN inhibitor resistance are consistent with the *EP300*-mutant *GATA6*-low cell lines we describe here. Based on our observation that Wnt inhibition drives an increase in cancer cell–derived Wnt production (Figure 8C), we propose instead that the elevated expression of several Wnts in the *GATA6*-low pancreatic tumors (Figure 8A) is due to a compensatory feedback response, reacting to the loss of Wnt/β-catenin signaling.

Both p300 and CBP have diverse targets and are well-known coactivators of the β-catenin/TCF transcriptional machinery. However, our short-term *EP300* knockout did not decrease the expression of β-catenin target genes (Figure 4D), reflecting perhaps the redundancy with CBP. The loss of Wnt/β-catenin activity seen in the existing *RNF43*/*EP300*-mutant cancer cell lines (Figure 1H and Figure 4G) is likely due to the loss of any selective pressure to maintain Wnt signaling in these cells once p300 is mutated. Both p300 and CBP share high-sequence homology and regulate many, but not all, target genes redundantly (77). Interestingly, only p300, but not CBP, was identified from our CRISPR screens and was inactivated in the existing PORCN inhibitor–resistant cell lines, highlighting a specific role of p300 in determining Wnt dependency. Determining why CBP cannot replace p300 in sustaining *GATA6* expression requires additional study. The transcription factor or factors that recruit p300 (but perhaps not CBP) to the *GATA6* locus await further characterization.

While active Wnt signaling is found in many cancers (78), our data suggest there may be a selective advantage to bypassing Wnt signaling during pancreatic cancer evolution. For example, in the Patu8988T orthotopic xenograft model, Wnt inhibition even slightly promoted tumor growth (Figure 1B). The situation may be similar in colorectal cancers, where β-catenin activation via *APC* mutation is common. The subtype of colorectal cancers (CMS2) with the highest Wnt-signaling activation shows epithelial differentiation and has the best clinical outcome, whereas the subtype (CMS4) with the lowest Wnt signature has mesenchymal and metastatic features and the poorest prognosis (79). Likewise, in a model system, LGR5-negative (i.e., with low Wnt signaling) colorectal cancer cells with high plasticity were found to drive metastasis (80). Thus, while active Wnt signaling maintains the stemness of cancer cells, it may also restrict them to the epithelial lineage. Evading the dependency on Wnt signaling, for example, by bypassing the intrinsic GATA6-mediated differentiation program might drive cancer progression, invasion, and metastasis.

The roles of GATA6 in regulating differentiation in normal pancreas and pancreatic cancer are well established (48–53). How *GATA6* expression is regulated is less well understood. The *GATA6* genomic locus is hypermethylated in the *GATA6*-low basal-like pancreatic tumors (7), although this may be a late event that reinforces rather than establishes *GATA6* silencing (81–83). Here, we report that p300 directly regulates *GATA6* transcription. Loss of p300 downregulated the abundance of H3K27Ac at the *GATA6* promoter and an intronic enhancer and decreased *GATA6* expression by approximately 50%. This decrease in *GATA6* mRNA largely blocked GATA6 activity (Figure 5B), which could be due to the reported sensitivity of GATA6 to haploinsufficiency (49, 53). Notably, given that the frequency of genetic alterations in *EP300* is less than 2% in treatment-naive pancreatic tumors, there are likely to be additional mechanisms regulating *GATA6* expression in basal-like tumors that may be dependent or independent of p300.

It is notable that despite changes in *GATA6* expression between the classical and basal-like subtypes, there is no change in the *EP300* mRNA level (Supplemental Figure 18A). We speculate that the above-mentioned hypermethylation of the *GATA6* genomic locus renders that locus insensitive to p300. While we focused on GATA6 in this study, several other lineage-related transcription factors (68), e.g., EHF, ELF3, HNF4A, and KLFs, likely also contributed to the differentiation regulation and formed an interconnected regulatory network together with GATA6 (Figure 5A and Figure 7E) to control subtype transition.

Last but not least, our findings provide a rationale for using p300 and GATA6 expression status to further stratify patient selection in *RNF43*-mutant pancreatic cancers to maximize the clinical efficacy of Wnt inhibitors. The loss of the p300/GATA6 axis, by causing dedifferentiation, may cause resistance to other chemotherapeutic agents as well. In addition, complementary to the reported synthetic lethality of targeting p300 in *CREBBP*-mutant cancer cells (84), the synthetic lethality of targeting CBP in *EP300*-mutant cases offers a potential alternative therapeutic opportunity for these patients.

## Methods

Please refer to Supplemental Methods for experimental details. The raw RNA-Seq data have been deposited in the NCBI’s Gene Expression Omnibus database (GEO GSE183893).

### Statistics.

Statistical analysis was performed using R or GraphPad. A *P* value of less than 0.05 was considered significant. For CRISPR screen and RNA-Seq analysis, an FDR of less than 10% was considered significant. For transcription regulator binding enrichment analysis, an FDR of less than 5% was considered significant.

### Study approval.

The SingHealth Institutional Animal Care and Use Committee approved all animal studies, which complied with applicable regulations.

## Author contributions

ZZ, BM, and DMV designed the study. KCW advised on library design and contributed the cancer-focused CRISPR library. ZZ and BM performed the animal studies. ZZ performed the biochemical analysis. ZZ and NH performed the bioinformatics analyses. BM and DMV supervised the study. ZZ and DMV wrote the first draft of the manuscript, which was reviewed, revised, and approved by all of the authors.

## Supplementary Material

Supplemental data

Supplemental table 1

Supplemental table 2

Supplemental table 3

Supplemental table 4

## Version 1 05/10/2022

In-Press Preview

## Version 2 06/15/2022

Electronic publication
