# Supplementary material for: A p300/GATA6 axis determines differentiation and Wnt dependency in pancreatic cancer models
Source: J Clin Invest. 2022 Jun 15;132(12):e156305. doi: 10.1172/JCI156305 (PMC9197518; doi:10.1172/JCI156305)
Supplement: Supplemental data [file jci-132-156305-s049.pdf]

Supplementary Materials

A p300/GATA6 axis determines differentiation and Wnt dependency in pancreatic cancer models

Zheng Zhong, Nathan Harmston, Kris Wood, Babita Madan, David M. Virshup

- **Supplementary Methods**
- **Supplementary References**
- **Supplementary Figure S1-18 and legends**
- **Supplementary Table S1-4 (separate files)**

Supplementary Methods

Cell lines

HPAF-II, CFPAC-1, and Panc10.05 cells were from Duke Cell Culture Facility. AsPC-1, Capan-2, and PL45 cells were from ATCC. Patu8988S and Patu8988T were from DSMZ. HPAF-II cells were grown in Eagle's Minimum Essential Medium (EMEM) with 10% FBS. CFPAC-1 cells were grown in Iscove's Modified Dulbecco's Medium (IMDM) with 10% FBS. PL45, Patu8988S, and Patu8988T cells were grown in Dulbecco's Modified Eagle's Medium (DMEM) with 10% FBS. AsPC-1 cells were grown in Roswell Park Memorial Institute (RPMI) 1640 Medium with 10% FBS. Capan-2 cells were grown in RPMI 1640 with 20% FBS. Panc10.05 cells were grown in RPMI 1640 with 10% FBS and 0.1% (v/v) insulin (Gibco, #12585014). All culture media contained 1 mM sodium pyruvate, 2 mM L-glutamine, and 1% (v/v) penicillin/streptomycin (Gibco, #15140122). Cells were cultured in a humidified incubator with 5% CO_2. All cell lines were regularly tested for mycoplasma contamination and confirmed to be mycoplasma free.

Plasmids

lentiCRISPR v2 (Addgene #52961) was a gift from Feng Zhang (98). pMD2.G (Addgene #12259) and psPAX2 (Addgene #12260) were gifts from Didier Trono. pcDNA3.1-p300 (Addgene #23252) and pcDNA3.1-300(HAT-) (Addgene #23254) were gifts from Warner Greene (99).

sgRNAs listed in the following table were cloned into lentiCRISPR v2 (Addgene #52961) following protocols described in previous studies (98, 100).

sgRNA	Sequence (5'-3')
sgNTC #1	GACCGGAACGATCTCGCGTA
sgEP300 #2	ACAGAATTGGGACTAACCAA
sgEP300 #5	TCAGATGCCGACACAACCCC
sgCREBBP #1	GATGAGCTGATACCCAATGG
sgCREBBP #3	GAAGACGGAGACCCAAGCAG
sgGATA6 #2	GGGCAGCATGGAACCCACGC
sgGATA6 #5	GGTGGTGGGCACGTAGACCG

To construct the $GATA6_S$ expressing plasmid, *GATA6* cDNA was synthesized using HPAF-II total RNA as template, primer 5'-CAAAAGCAGACACGAGTGGA-3', and RevertAid First Strand cDNA Synthesis Kit (Thermo Scientific, K1621). The $GATA6_S$ sequence was PCR amplified from the cDNA using Phusion Hot Start Flex Master Mix (NEB,

M0536S) with 5% DMSO (v/v) and cloned into the pLenti6/V5-D-TOPO vector (Invitrogen, K4955-10).

To construct the luciferase reporting plasmids, the *GATA6* promoter (101) and enhancer regions were PCR amplified using HPAF-II genomic DNA as template. Primers 5’-TTCTCTGGCCTAACTGGCCGGTACCACGCCTCTTGTCCTAAAGTCTC-3’ and 5’-CCGCCGAGGCCAGATCTTGATATCCCGAGCCCTAAACAAACAGC-3’ were used for amplifying the promoter region. Primers 5’-CTACAAATGTGGTAAAATCGATAAGGATCTCTTGCCTCAGGATTCTGTC-3’ and 5’-CTCAAGGGCATCGGTCGACGTACCAGAAACACAAGGCAC-3’ were used to amplify the enhancer region. The promoter amplicon was purified and inserted into KpnI-HF (NEB, R3142S) and XhoI (NEB, R0146S) double digested pGL4.10[*luc2*] vector (Promega, E6651) using NEBuilder HiFi DNA Assembly Master Mix (NEB, E2621S) to get the pro-Luc plasmid. The KpnI-HF and XhoI double digested pGL4.10[*luc2*] vector was ligated using oligo 5’-TTCTCTGGCCTAACTGGCCGGTACCGGATATCAAGATCTGGCCTCGGCGG-3’ and NEBuilder HiFi DNA Assembly Master Mix to get the empty vector control (EV-Luc). The enhancer amplicon was purified and inserted into the BamHI-HF (NEB, R3136S) digested pro-Luc plasmid using NEBuilder HiFi DNA Assembly Master Mix to get the pro-Luc-en plasmid.

Lentivirus

For lentivirus packaging, the lentiviral vector was co-transfected with pMD2.G (Addgene #12259) and psPAX2 (Addgene #12260) into HEK293FT cells using Opti-MEM (Gibco, #31985070), lipofectamine 2000 (Invitrogen, #11668019), and PLUS reagent (Invitrogen, #11514015). The Opti-MEM was replaced with DMEM six hours post transfection. The virus-containing culture media were collected 48 hours and 72 hours post transfection, filtered through 0.45 μm filter membrane, and stored at -80 °C. Virus titration was performed for the CRISPR lentivirus libraries by serial dilution of the virus-containing media for transduction and measuring the percentage of viable cells after puromycin selection using CellTiter-Glo Luminescent Cell Viability Assay (Promega, G7570).

Animal studies

Cancer xenograft experiments were performed in NOD scid gamma (NSG) mice purchased from InVivos, Singapore or Jackson Laboratories (NOD.Cg-$Prkdc^{scid}$ $Il2rg^{tm1Wjl}$/SzJ, catalog #005557), Bar Harbor, Maine and bred and housed in specific pathogen-free conditions.

For orthotopic implantation, cells stably expressing firefly luciferase were harvested using TrypLE (Gibco, #12605028), washed with cold PBS, and resuspended in ice cold 50% Matrigel (Corning, #354248) in PBS. 2 × 10^6 (HPAF-II) or 5 × 10^6 (Patu8988S or Patu8988T) cells in ~50 µl 50% Matrigel were injected into the pancreas of NSG mice. Orthotopic tumors in the living animals were monitored weekly by injecting 150 mg/kg D-luciferin (PerkinElmer, #122799) intraperitoneally into the mice and imaging with a PerkinElmer In Vivo Imaging System (IVIS).

For subcutaneous implantation, cells were resuspended in ice cold 50% Matrigel in PBS. 5 × 10^6 (for drug efficacy studies) or 1 × 10^7 (for *in vivo* CRISPR screens) cells in ~200 µl 50% Matrigel were injected into the flank of NSG mice. Tumor dimensions were periodically measured using a caliper and tumor volumes were calculated as 0.5 × length × $(\text{width})^2$.

ETC-159 was formulated in 50% PEG 400 (v/v) in water and administered by oral gavage at a dosing volume of 10 µl/g mice body weight once every day (q.d.) or once every other day (q.o.d.). Mice were sacrificed 6 h (or 8 h for RNA-seq study) after the last dosing. The tumors and organs with metastases, if any, were harvested and snap frozen in liquid nitrogen followed by storage at -80 °C, or immediately fixed in 10% neutral buffered formalin followed by paraffin embedding.

In vivo CRISPR screens

Four ~200-gene CRISPR libraries were constructed following published methods (26, 86). The sgRNA sequences were selected from a pre-designed genome-wide CRISPR library (86) with five sgRNAs per gene. Each library also contained sgRNAs targeting core essential genes (*PCNA*, *POLR2A*, *PSMA7*, *RPS27*, and *SF3A3*), known Wnt pathway genes, and 50 non-targeting control sgRNAs. Oligonucleotides had the structure: 5'-library-specific tag + 5'-adaptor + sgRNA sequence + 3'-adaptor + 3'-library-specific tag and were synthesized by CustomArray (Redmond, WA, USA). They were amplified using nested PCR to get four sub-pools with the structure: 5'-homology arm + sgRNA sequence + 3'-homology arm. These PCR products were gel purified and cloned into BsmBI-digested lentiCRISPRv2 vector (Addgene #52961, where the sgRNA sequence and Cas9 protein are encoded by the same vector) by Gibson assembly (NEB, E2611S). The assembly products were electroporated into Endura ElectroCompetent cells (Lucigen, 60242-2) for amplification followed by plasmid midiprep. The fifth CRISPR library that targets 378 genes was described previously (18, 26).

For each CRISPR library, the plasmid was packaged into lentivirus. HPAF-II cells were transduced with this lentivirus library at MOI = 0.3 in the presence of 7.5 μg/ml polybrene. 2 × 10^6 HPAF-II cells were used for transduction of each CRISPR library with ~1,000 or 2,000 sgRNAs to ensure the library coverage of ~600 or 300 cells/sgRNA given the MOI. The transduced HPAF-II cells were selected with 0.8 μg/ml puromycin for 7 days and expanded *in vitro* for another 3-7 days. This expanded HPAF-II pool was injected subcutaneously into the flank of NSG mice. 10^7 cells were injected per flank to get library coverage of 10,000 or 5,000 cells/sgRNA. 10^6 cells were collected just before injection and stored as a cell pellet at -80 °C prior to deep sequencing (Fig 2A). Mice were sacrificed following vehicle or ETC-159 treatment and tumors were harvested and stored at -80 °C. The integrated sgRNA cassette was amplified from the DNA of library plasmids, lentiviruses, and HPAF-II cells and tumors by PCR as described (18, 26). Illumina sequencing adaptors and index sequences were added onto the PCR products from each sample by a second round of PCR. The barcoded PCR products were purified using the QIAGEN Gel Purification Kit, quantified using the KAPA Library Quantification Kit, and sequenced on an Illumina MiSeq platform.

The MiSeq data were analyzed as described previously (18). Briefly, the fastq file was processed by R. The "processAmplicons" package from the "edgeR" library (87) was used to decode samples, align the sequences, and count the read number of each sgRNA in each sample. In each sample, the read counts of each sgRNA were normalized to the read count sum of non-targeting control sgRNAs. MAGeCK(88) was used to compare different groups of samples with each other and identify the statistically significantly depleted or enriched sgRNAs and the corresponding genes.

CRISPR genome editing

To generate *EP300*, *CREBBP*, or *GATA6* knockout cells, sgRNAs were cloned into lentiCRISPR v2 (Addgene #52961) and packaged as lentivirus. Cells were transduced with the lentivirus and selected with puromycin. To analyse the genome editing, genomic DNA of the puromycin-selected cells was extracted and PCR amplified using primers surrounding the expected cutting site. The PCR products were purified and subject to Sanger sequencing. Indels were visualized using TIDE (89). To establish single cell-derived subclones, the puromycin-selected cells were sorted into 96-well plates as single cell per well using flow cytometry. Cells were maintained in 96-well plates with regular culture media changing. When the cells reached > 50% confluence in a well, ~50% of cells in the well were passaged into a well of 12-well plate

and the remaining cells in the well were directly lysed (90) to get crude genomic DNA for PCR and Sanger sequencing as described above. Subclones with frameshift indels were expanded and maintained.

RNA-seq

NSG mice bearing orthotopic HPAF-II *EP300* wildtype or knockout tumors were treated with vehicle or ETC-159 (5 or 15 mg/kg q.d.) for three days and tumors were harvested 8 hours after the third dose. Total RNA isolated from these tumors underwent ribosomal RNA depletion followed by stranded total RNA library prep and sequencing on an Illumina HiSeq 3000 platform (150 × 2 bp). The raw RNA-seq data have been deposited in the NCBI's Gene Expression Omnibus (GEO) database under GSE183893.

Sequenced reads were assessed for quality using FastQC. Reads originating from mouse (mm10) were removed using Xenome (91). The remaining reads were aligned against hg38 (Ensembl version 79) using STAR (92) and quantified at the gene-level using RSEM (93). Reads mapping to chrM or genes which were annotated as *rRNA* or *Mt_rRNA* were removed. In addition, reads mapping to *RN7SK*, *RN7SL2*, *RN7SL1* and *Nuclear RNase P* were filtered out. Only those genes that were expressed at more than 1 TPM in at least one condition were used in this analysis. Differential expression analysis was performed using DESeq2 (94). Independent filtering was not used in this analysis. Pairwise comparisons were performed using a Wald test. To control for false positives due to multiple comparisons in differential expression analysis, we empirically calculated the false discovery rate (FDR) using fdrtool (95).

The transcription regulator binding enrichment analysis for the differentially expressed genes (~2600 genes, FDR < 0.01) in vehicle-treated *EP300* knockout HPAF-II orthotopic tumors compared to vehicle-treated *EP300* wildtype tumors was performed using Enrichr and the ChEA_2016 library (46, 47).

The expression levels (TPM) of human WNT genes in HPAF-II orthotopic tumors in response to ETC-159 treatment were derived from our previously published RNA-seq dataset (17). Expression levels (TPM) of mouse stromal genes was calculated by using only those reads classified as 'host' (i.e. originating from mouse) from that RNA-seq dataset. These reads were mapped to mm10 (Ensembl version 79) and quantified using STAR and RSEM, respectively.

Histological analysis

Formalin-fixed paraffin-embedded tissue sections were deparaffinized and rehydrated. Hematoxylin and Eosin (H&E) staining was performed following the standard protocol. Alcian

Blue (pH 2.5) mucin staining was performed using the Alcian Blue (pH 2.5) Mucin Stain Kit (Abcam, ab150662) following the manufacture's protocol. Ki67 or β-catenin staining was performed similarly as described in previous studies (96). Anti-Ki67 antibody (Leica, KI67-MM1-L-CE) and anti-β-catenin antibody (BD, #610154) were used. The stained sections were dehydrated and mounted using DPX. Bright field images were taken using a Nikon ECLIPSE Ni-E microscope. QuPath was used for staining quantification (97).

***In vitro* drug sensitivity assays**

For the 2D culture assay, cells were seeded in 24-well plate or 48-well plate at low density (1,000 cells/well). The next day, media in the wells were replaced with fresh media containing DMSO control or ETC-159 dissolved in DMSO. The final concentration of DMSO in all the wells was 0.1% v/v. The media were changed with fresh media containing DMSO or ETC-159 regularly. When cells in the DMSO control wells reached confluency, all the media were removed and cells were washed with PBS, fixed by ice cold methanol, and subsequently stained with crystal violet following standard protocol. For quantification purpose, the crystal violet dye was released from the stained cells by adding 250 μl 1% sodium deoxycholate solution to each well and incubating them at room temperature for 3-4 hours with gentle shaking. The crystal violet dissolved in sodium deoxycholate solution was transferred to a 96-well plate and the optical density (OD) values at 595 nm were read using a Tecan Infinite M200 plate reader.

Soft agar colony formation assays were performed in 48-well suspension culture plates. 0.6% (g/ml) agarose (Sigma, A9045) in 200 μl complete culture media was layered on the bottom of each well. After solidification of the bottom agarose layer at room temperature, 3,000 cells/well mixed with 0.36% (g/ml) agarose in 200 μl complete culture media were layered on top of that. The plates were then incubated at 4 °C for 1 hour to let the cell/agarose layer solidify. Subsequently, 450 μl complete culture media containing DMSO or ETC-159 were added to each well. Cells were allowed to form colonies and grow for 1-2 weeks in an incubator. The colonies were then stained with MTT (3-(4,5-dimethylthiazol-2-yl)-2,5-diphenyltetrazolium bromide) and counted by GelCOUNT (Oxford Optronix, Abingdon, UK).

DNA/RNA isolation and quantitative PCR (qPCR)

Genomic DNA from fresh cells, frozen cell pellets or frozen tumor tissues was extracted using a salting out protocol. Briefly, the frozen tumor tissues were homogenized in liquid nitrogen with a mortar and pestle. Cells or homogenized tissues were resuspended in a lysis

buffer containing 10 mM Tris, pH 7.5, 400 mM NaCl, 100 mM EDTA, and 0.6% SDS, followed by digestion with Proteinase K and RNase A. Proteins were precipitated by adding saturated NaCl solution. The supernatant was collected and genomic DNA was precipitated by ethanol.

In CRISPR screens, the viral RNA was extracted using the QIAamp Viral RNA Kit and reverse transcribed into cDNA using Thermo Scientific RevertAid First Strand cDNA Synthesis Kit and viral specific primers.

Total RNA from fresh cells or frozen tumor tissues was isolated using the QIAGEN RNeasy Kit. Total RNA was reverse transcribed into cDNA using the Bio-Rad iScript cDNA synthesis Kit. Real time qPCR was performed using the Bio-Rad SsoFast EvaGreen Supermix or SsoAdvanced Universal SYBR Green Supermix on Bio-Rad CFX96 Touch real-time PCR machine. Primer sequences are described in the following table.

Gene	Forward primer (5'-3')	Reverse primer (5'-3')
AXIN2	CAACACCAGGCGGAACGAA	GCCCAATAAGGAGTGTAAGGACT
ACTB	AAGGATTCCTATGTGGGCGACG	GCCTGGATAGCAACGTACATGG
EP300	AACAGCAGCTCAACCATCCA	TCCGGCGTAGGAAATATGGC
GATA6	CTGCGGGCTCTACAGCAAG	GTTGGCACAGGACAATCCAAG
GAPDH	GGAGCGAGATCCCTCCAAAAT	GGCTGTTGTCATACTTCTCATGG
LGR5	CACCTCCTACCTAGACCTCAGT	CGCAAGACGTAACTCCTCCAG
NKD1	TCGCCGGGATAGAAAACTACA	CAGTTCTGACTTCTGGGCCAC
NOTUM	AACGCAAACATGGTCTTCATCC	GGCGTACTCGTTCTTCTCAGA

Western blot

Fresh cells or frozen tumor tissues were homogenized in 4% SDS in water using a tissue homogenizer. Protein concentration of the lysate was quantified using the BCA assay. Equal amounts of total proteins for different samples were resolved by 10% SDS-PAGE or 4-20% SDS-PAGE (Bio-Rad, #4561094) for blotting p300 and histone H3 followed by wet transfer to PVDF membranes. Western blot was performed following standard methods. Primary antibodies used for Western blot included anti-p300 (Abcam, #10485), anti-H3K27Ac (Abcam, #4729), anti-histone H3 (CST, #4499), anti-GATA6 (CST, #5851), anti-β-actin (CST, #4967). The SuperSignal West Dura Extended Duration Substrate (Thermo Scientific, #34075), SuperSignal West Femto Maximum Sensitivity Substrate (Thermo Scientific, #34096), or Cytiva Amersham ECL Select Western Blotting Detection Reagent (Fischer Scientific, #45000999) were used for developing the blots. The images were captured digitally using the

Bio-Rad ChemiDoc Touch Imaging system. The Restore Western Blot Stripping Buffer (Thermo Scientific, #21059) was used when stripping and reprobing were required.

Dual-luciferase reporter assay

HEK293 cells were seeded in 24-well plates. In each well, cells were transfected with equal molar amount of EV-Luc (100 ng), pro-Luc (128 ng), or pro-Luc-en (193 ng) plasmids and co-transfected with 1 ng pRL *Renilla* luciferase expression vector (Promega, E2231) ± 10 ng pcDNA3.1-p300 (Addgene #23252) or pcDNA3.1-300(HAT-) (Addgene #23254). Two days post transfection, the activity of firefly luciferase and renilla luciferase was measured using the Dual-Luciferase Reporter Assay System (Promega, E1960) following the manufacturer's protocol.

CUT&RUN assay

The CUT&RUN assay for H3K27Ac was performed in HPAF-II NTC or *EP300* knockout cells and Panc10.05 cells using the CUT&RUN Assay Kit (CST, #86652) following the manufacturer's protocol. 100,000 cells were used for each reaction. Anti-H3K27Ac antibody (Abcam, #4729) and rabbit IgG isotype control from the CUT&RUN Assay Kit were used. DNA was purified using phenol/chloroform and qPCR was performed as described above. Primer sequences are described in the following table.

Region	Forward primer (5'-3')	Reverse primer (5'-3')
GATA6 promoter	CGCAGCCTTATCTCCGAGTT	CTCTGCCTGCCTAACTACCG
GATA6 enhancer	GCAGTGAAGGAGGCATGTGA	GCTGAAGGACGACTGTGTGA

Public dataset analysis

Gene expression levels in pancreatic cancer cell lines were extracted from the Cancer Cell Line Encyclopedia (CCLE) database (23) or from Klijn et al. RNA-seq dataset (45).

Whole genome sequencing (WGS) data of Patu8988T cells (SRA accession number: SRX5466648) from the CCLE study (23) was visualized using the Integrative Genomics Viewer (IGV) (102).

Gene dependency scores (Bayes Factors) of *EP300* and *CREBBP* in a panel of pancreatic cancer cell lines based on *in vitro* CRISPR screens (103) were extracted from the PICKLES database (104) in February 2021.

The GATA6 ChIP-seq data in Patu8988S cells (GEO accession number: GSE47535) (60), UCSC conservation track (100 vertebrate species), ENCODE p300 ChIP-seq tracks (ENCSR000EGE; ENCSR000BLW), ENCODE layered H3K27Ac ChIP-seq track (7 cell lines), ENCODE layered H3K4Me1 ChIP-seq track (7 cell lines), H3K27Ac ChIP-seq data in a panel of pancreatic cancer cell lines (GEO accession number: GSE64557) (105), and ENCODE H3K27Ac ChIP-seq tracks for mouse intestines (106) were visualized in the UCSC genome browser (107).

Gene expression levels in MCF7 cells ± *EP300* knockdown were derived from Asaduzzaman *et al*. microarray dataset (GEO accession number: GSE76200) (108).

Gene expression levels in a cohort of resected treatment-naïve pancreatic tumors were derived from the ICGC study (7). Gene expression levels in pancreatic primary tumors and matched liver metastases from same patients were derived from Yang *et al*. RNA-seq dataset (GEO accession number: GSE151580) (76). The median expression level of each Wnt in pancreatic tumors of the TCGA cohort and normal pancreatic tissues from TCGA and GTEx databases was extracted through the GEPIA website (109). Single-cell RNA-seq results of primary pancreatic tumors (73) were visualized with the TISCH website (110).

Supplementary References

86. Wang T, Wei JJ, Sabatini DM, Lander ES. Genetic screens in human cells using the CRISPR-Cas9 system. *Science* 2014;343(6166):80–84.

87. Dai Z et al. edgeR: a versatile tool for the analysis of shRNA-seq and CRISPR-Cas9 genetic screens. *F1000Res* 2014;3:95.

88. Li W et al. MAGeCK enables robust identification of essential genes from genome-scale CRISPR/Cas9 knockout screens. *Genome Biol.* 2014;15(12):554.

89. Brinkman EK, Chen T, Amendola M, van Steensel B. Easy quantitative assessment of genome editing by sequence trace decomposition. *Nucleic Acids Res.* 2014;42(22):e168.

90. Ramlee MK, Yan T, Cheung AMS, Chuah CTH, Li S. High-throughput genotyping of CRISPR/Cas9-mediated mutants using fluorescent PCR-capillary gel electrophoresis. *Sci Rep* 2015;5:15587.

91. Conway T et al. Xenome--a tool for classifying reads from xenograft samples. *Bioinformatics* 2012;28(12):i172–8.

92. Dobin A et al. STAR: ultrafast universal RNA-seq aligner. *Bioinformatics* 2013;29(1):15–21.

93. Li B, Dewey CN. RSEM: accurate transcript quantification from RNA-Seq data with or without a reference genome. *BMC Bioinformatics* 2011;12(1):323–16.

94. Love MI, Huber W, Anders S. Moderated estimation of fold change and dispersion for RNA-seq data with DESeq2. *Genome Biol.* 2014;15(12):550–21.

95. Strimmer K. fdrtool: a versatile R package for estimating local and tail area-based false discovery rates. *Bioinformatics* 2008;24(12):1461–1462.

96. Proffitt KD et al. Pharmacological inhibition of the Wnt acyltransferase PORCN prevents growth of WNT-driven mammary cancer. *Cancer Res.* 2013;73(2):502–507.

97. Bankhead P et al. QuPath: Open source software for digital pathology image analysis. *Sci Rep* 2017;7(1):16878.

98. Sanjana NE, Shalem O, Zhang F. Improved vectors and genome-wide libraries for CRISPR screening. *Nature Methods* 2014;11(8):783–784.

99. Chen LF, Mu Y, Greene WC. Acetylation of RelA at discrete sites regulates distinct nuclear functions of NF-kappaB. *The EMBO Journal* 2002;21(23):6539–6548.

100. Shalem O et al. Genome-scale CRISPR-Cas9 knockout screening in human cells. *Science* 2014;343(6166):84–87.

101. Sun Z, Pang S, Cui Y, Yan B. Genetic and Functional Variants Analysis of the GATA6 Gene Promoter in Acute Myocardial Infarction. *Front Genet* 2019;10:1100.

102. Robinson JT et al. Integrative genomics viewer. *Nat. Biotechnol.* 2011;29(1):24–26.

103. Behan FM et al. Prioritization of cancer therapeutic targets using CRISPR-Cas9 screens. *Nature* 2019;568(7753):511–516.

104. Lenoir WF, Lim TL, Hart T. PICKLES: the database of pooled in-vitro CRISPR knockout library essentiality screens. *Nucleic Acids Res.* 2018;46(D1):D776–D780.

105. Diaferia GR et al. Dissection of transcriptional and cis-regulatory control of differentiation in human pancreatic cancer. *The EMBO Journal* 2016;35(6):595–617.

106. Gorkin DU et al. An atlas of dynamic chromatin landscapes in mouse fetal development. *Nature* 2020;583(7818):744–751.

107. Kent WJ et al. The human genome browser at UCSC. *Genome Res.* 2002;12(6):996–1006.

108. Asaduzzaman M et al. Tumour suppressor EP300, a modulator of paclitaxel resistance and stemness, is downregulated in metaplastic breast cancer. *Breast Cancer Res Treat* 2017;163(3):461–474.

109. Tang Z et al. GEPIA: a web server for cancer and normal gene expression profiling and interactive analyses. *Nucleic Acids Res.* 2017;45(W1):W98–W102.

110. Sun D et al. TISCH: a comprehensive web resource enabling interactive single-cell transcriptome visualization of tumor microenvironment. *Nucleic Acids Res.* 2021;49(D1):D1420–D1430.

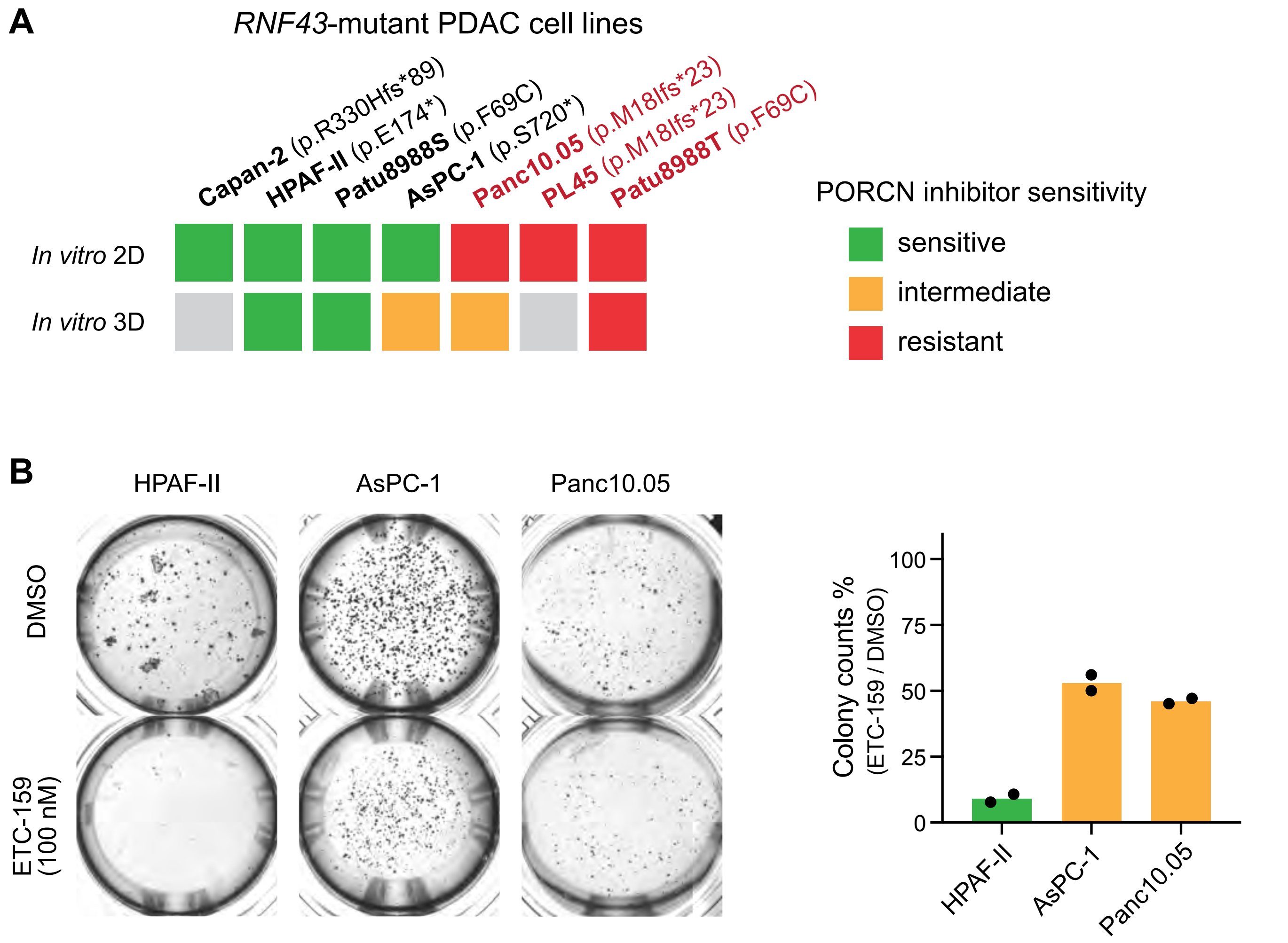

Supplementary Figure S1

Supplementary figure S1. Several *RNF43*-mutant pancreatic cancer cell lines are resistant or less sensitive to PORCN inhibitors.

(A) Summary of sensitivity of seven *RNF43*-mutant pancreatic cancer (PDAC) cell lines to PORCN inhibitors. *RNF43* mutations are shown in brackets. Sensitivity in 2D culture (*in vitro* 2D) is compiled from (5, 21); sensitivity in 3D culture (*in vitro* 3D) is compiled from **Fig. 1A** and **Supplementary Fig. S1B** (gray boxes: Capan-2 and PL45 did not form colonies in soft agar and so could not be scored). Sensitive, cell growth/colony formation inhibition > ~80%; intermediate, inhibition ≈ 50%; resistant, inhibition < ~20%.

(B) Sensitivity of HPAF-II, AsPC-1, or Panc10.05 cells to PORCN inhibitor ETC-159 in 3D culture. Cells were seeded in soft agar and treated with DMSO (0.1% v/v) or 100 nM ETC-159. Colonies were stained with MTT after ~two weeks of culture. Representative images are shown on the left and quantification of colony count is shown on the right (n = 2 biological replicates).

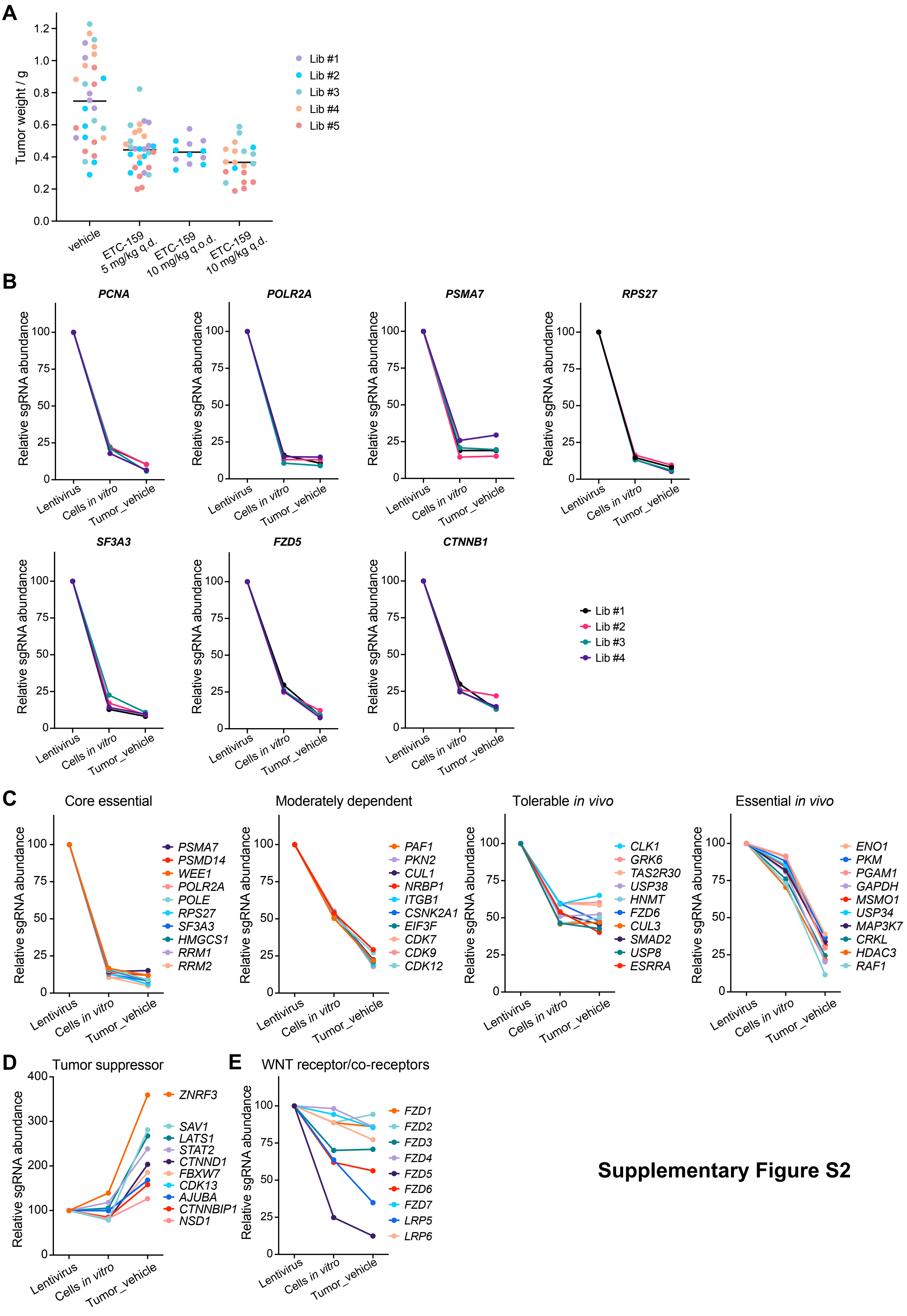

Supplementary Figure S2

Supplementary figure S2. *In vivo* CRISPR screens in a *RNF43*-mutant pancreatic cancer model.

(A) Tumor weight of the CRISPR library-carrying HPAF-II xenografts at the time of harvest. Each dot represents an individual tumor.

(B) Change of the abundance of sgRNAs targeting positive control genes in CRISPR library #1-4. The values indicated are the relative median abundance of all the five sgRNAs targeting a specific gene. The values are set to 100 for all genes in the lentivirus libraries.

(C) CRISPR screens classified fitness genes into different dependency patterns. For each class, the relative sgRNA abundance calculated as in **B** for 10 representative genes are shown.

Core essential: sgRNAs targeting genes involved in core biological processes, e.g. DNA/RNA polymerases and proteasome/ribosome/spliceosome subunits, were depleted quickly in the cell populations *in vitro* even before cells were injected into mice, reflecting that disruption in these biological processes markedly reduced cell fitness .

Moderately dependent: for some genes including several regulators of cell cycle (CDKs), their sgRNAs were depleted constantly but relatively slowly *in vitro* and *in vivo*, suggesting that their loss reduced the cell fitness to a certain extent, i.e. slowed down the cell cycle.

Tolerable *in vivo*: a group of genes whose sgRNA abundances only decreased *in vitro* but did not change obviously *in vivo*, suggesting that their loss might have an acute effect on the cell survival/proliferation in culture but could be tolerated later especially *in vivo*, potentially due to signaling rewiring, changed microenvironment or compensation.

Essential *in vivo*: a group of genes whose loss had no significant effect *in vitro* but clearly suppressed proliferation *in vivo*. These include genes involved in metabolism that we reported previously (18) and others whose mechanisms deserve further investigation.

(D) CRISPR screens identified tumor suppressors *in vivo*. 10 representative genes are shown. Notably, for most of these tumor suppressors, there was no obvious gain of fitness *in vitro* but disruption of tumor suppression function was clearly manifested by increased fitness during *in vivo* tumor growth.

(E) Change of the abundance of sgRNAs targeting Wnt receptors and co-receptors that are expressed in HPAF-II tumors.

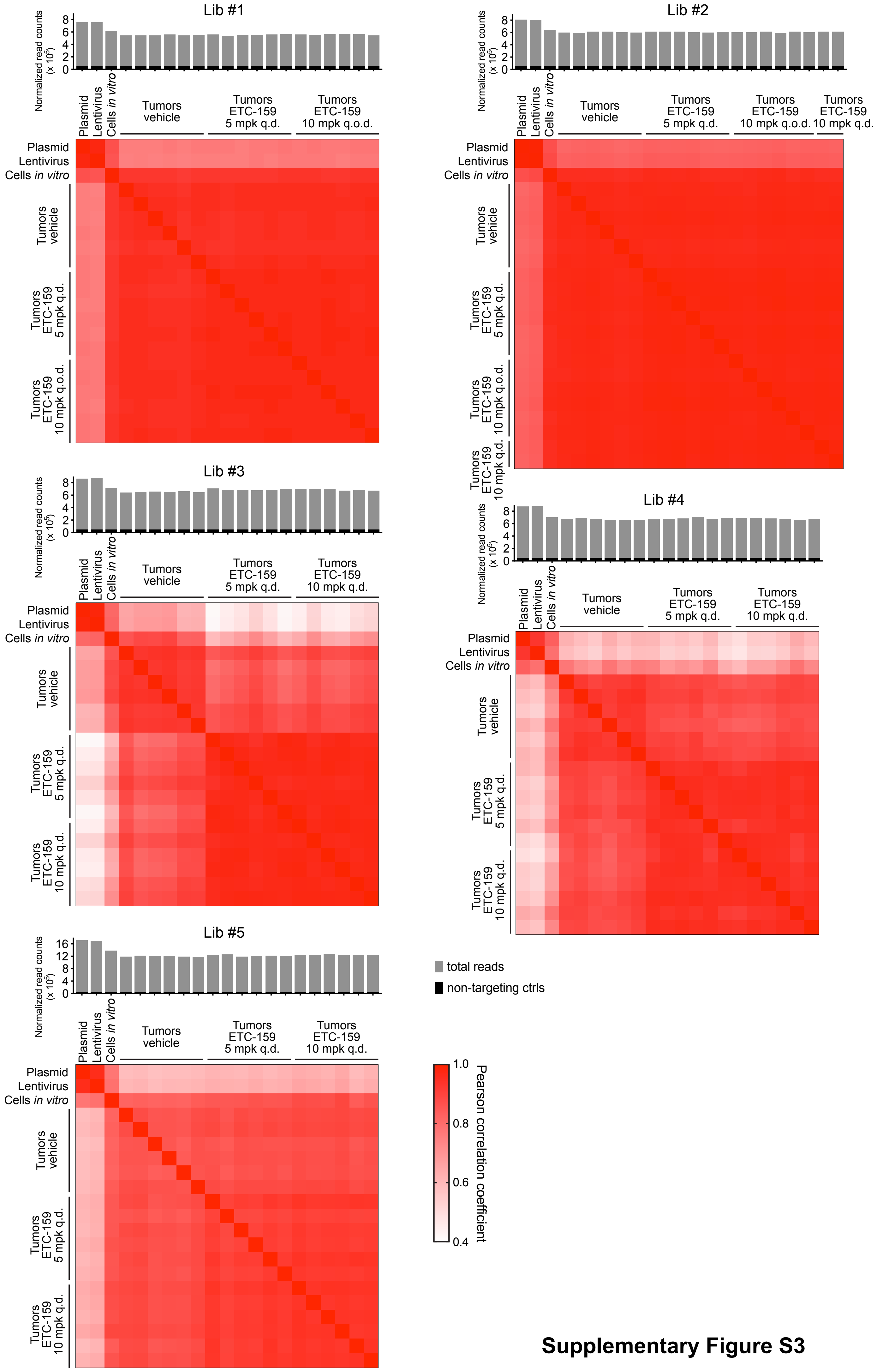

Supplementary Figure S3

Supplementary figure S3. High consistency of tumor replicates within each treatment group.

For each sample, the sum of read counts of all the sgRNAs normalized to the read counts of the nontargeting control sgRNAs from the respective samples is shown on the top. Pearson correlation analysis was performed for the normalized read counts of sgRNAs in all the samples and the Pearson correlation coefficients are shown as heatmap on the bottom of each panel.

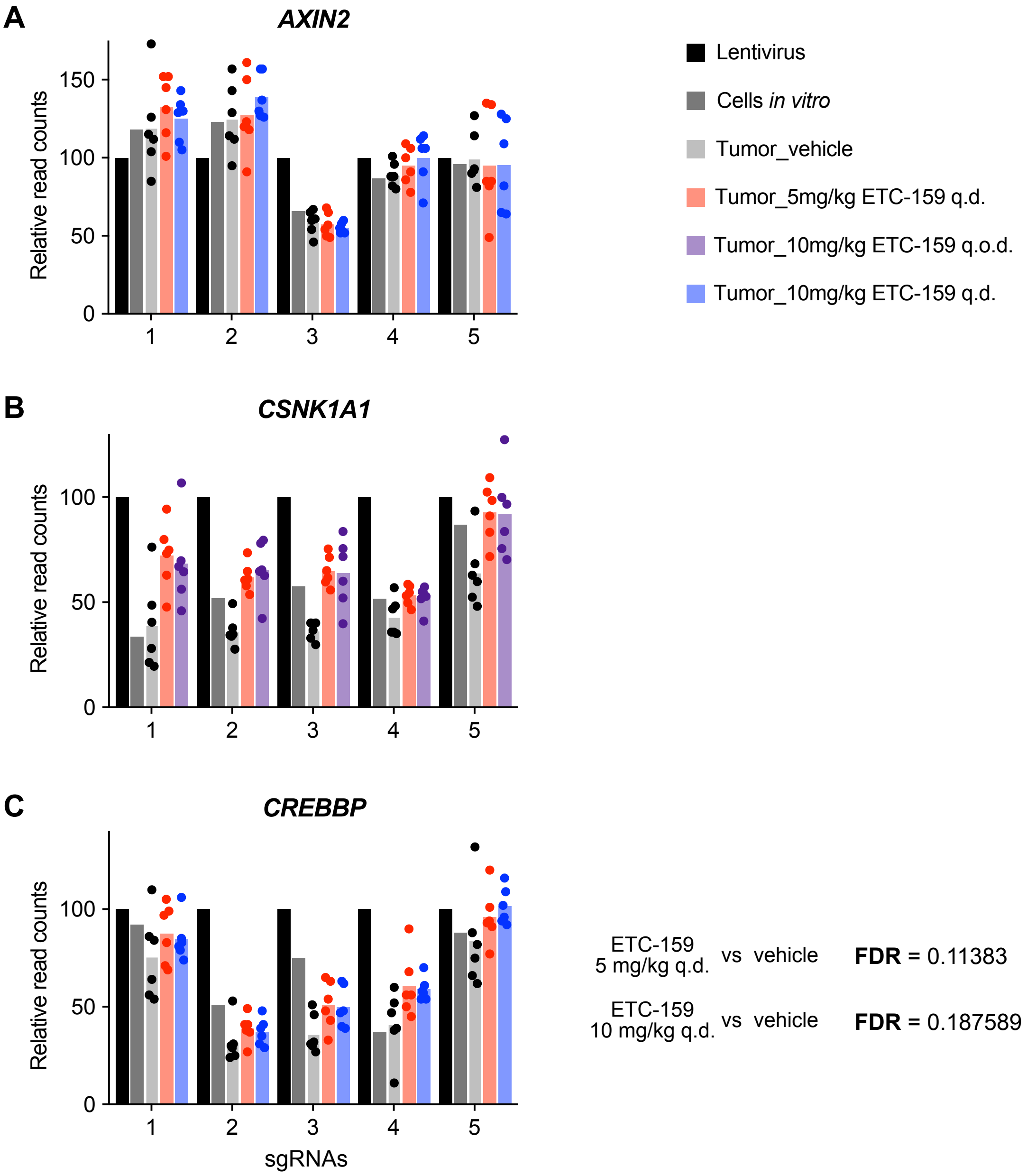

Supplementary Figure S4

Supplementary figure S4. Individual sgRNA read count change in all the samples for (A) *AXIN2*, (B) *CSNK1A1*, and (C) *CREBBP*.

For each gene, the five sgRNAs are shown. For each sgRNA, its read count in the lentivirus library is normalized to 100. Each dot represents the read count in an individual tumor (n = 6). For *CREBBP*, the gene level sgRNA enrichment FDRs calculated by MAGeCK are shown next to the graph.

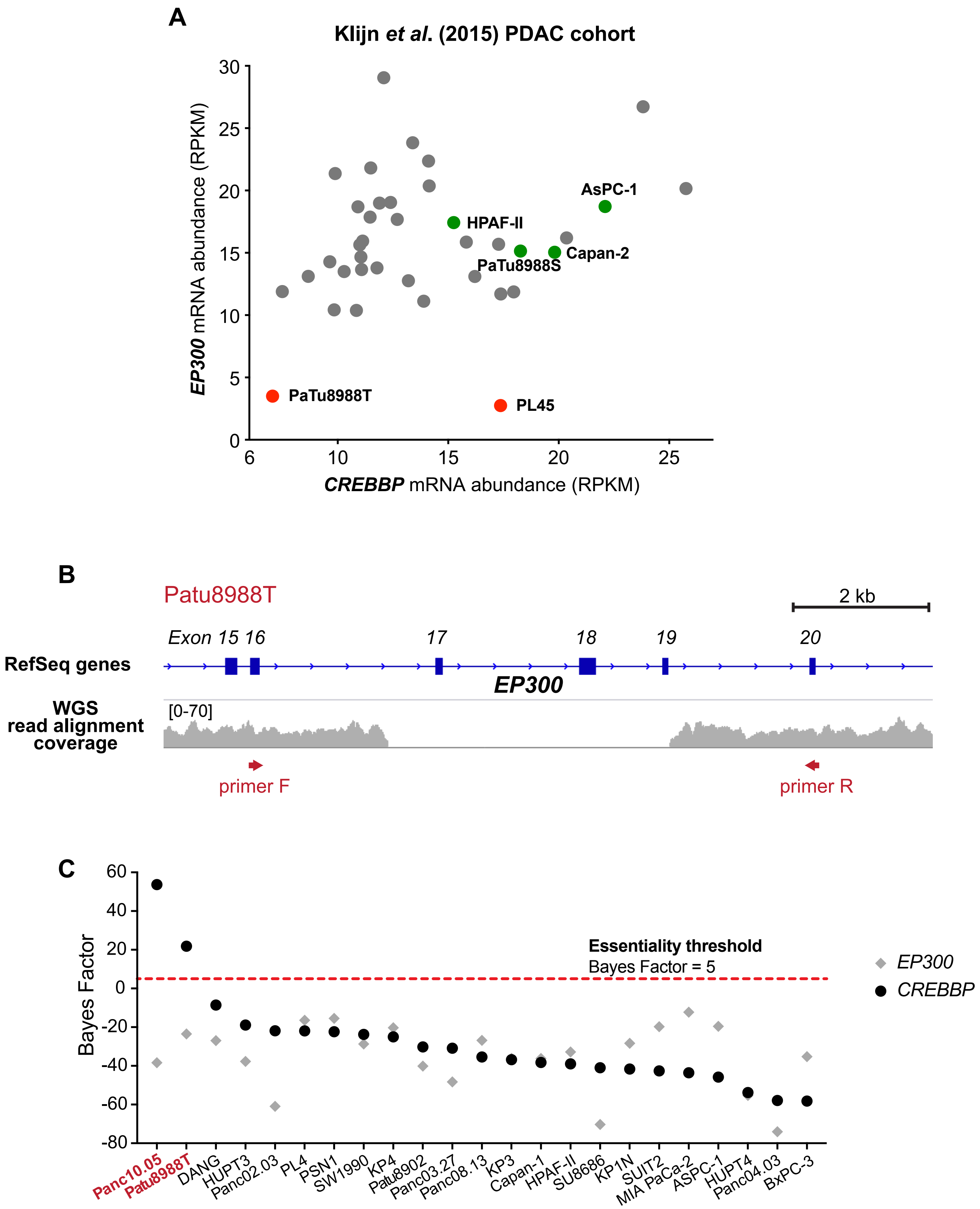

Supplementary Figure S5

Supplementary figure S5. PORCN inhibitor-resistant *RNF43*-mutant pancreatic cancer cell lines harbor loss-of-function genetic alterations in *EP300* and rely on *CREBBP*.

(A) mRNA abundances of *EP300* and *CREBBP* in a cohort of pancreatic cancer cell lines. The RPKM values of *EP300* and *CREBBP* were extracted from (45) for pancreatic cancer cell lines. Each dot represents an individual cell line. *RNF43*-mutant cell lines are labelled as green dots for PORCN inhibitor-sensitive ones and as red dots for resistant ones. Panc10.05 was not included in this dataset.

(B) A zoom-in view of the deleted region in the *EP300* locus in Patu8988T cells. The primers used in **Fig. 3D** are labelled in the graph.

(C) Synthetic lethality of *CREBBP* knockout in *EP300*-mutant pancreatic cancer cell lines. Dependency scores (Bayes Factors) of *EP300* and *CREBBP* in a panel of pancreatic cancer cell lines were extracted from the PICKLES database (104) in February 2021. Raw CRISPR screen data were reported in (103). Bayes Factor > 5 indicates essential genes.

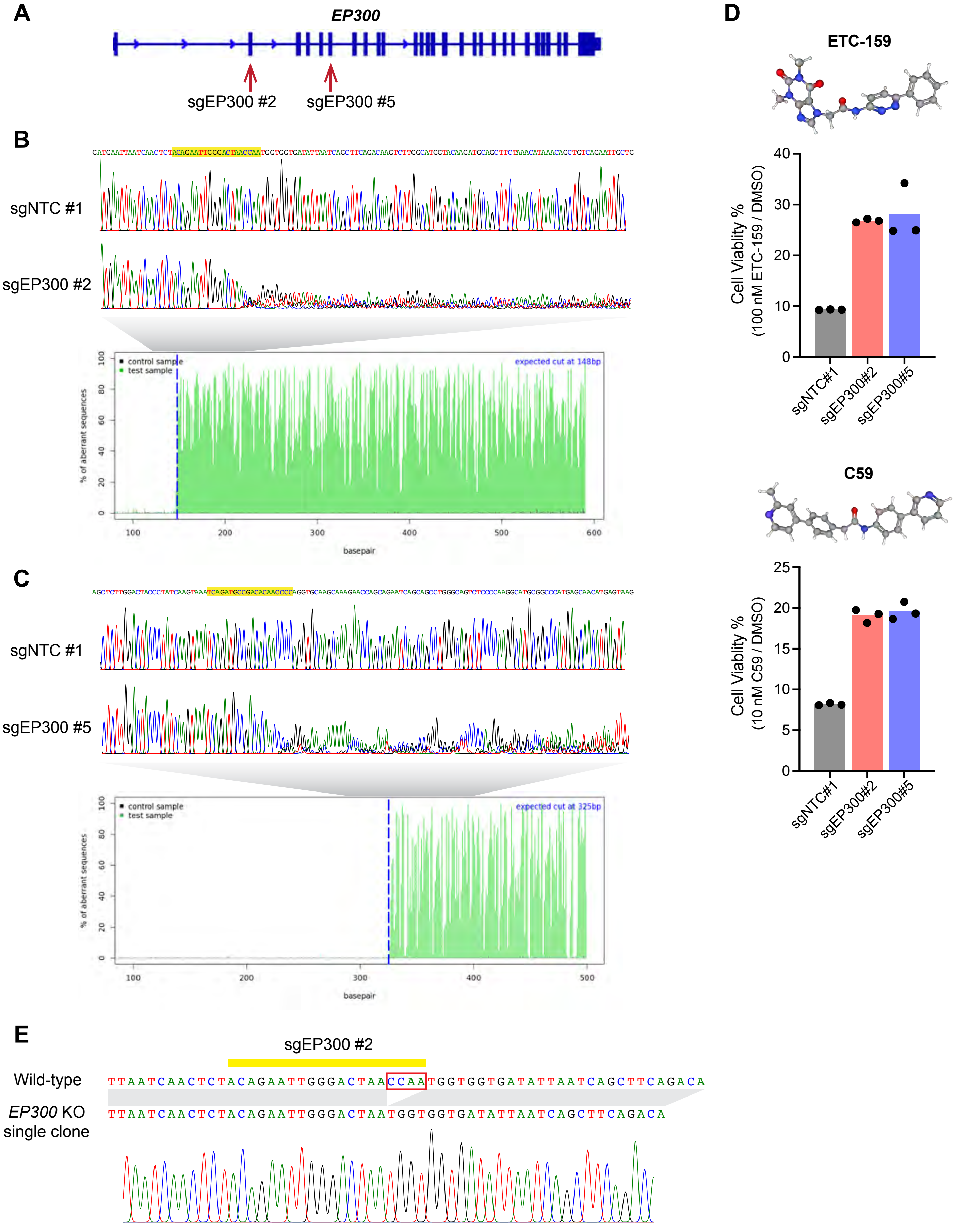

Supplementary Figure S6

Supplementary figure S6. *EP300* knockout in HPAF-II cells conferred resistance to PORCN inhibitors.

(A) Two individual sgRNAs were used to knockout *EP300*. The genome positions targeted by these two sgRNAs are labelled in the graph.

(B, C) Both sgRNAs led to considerable indels at the expected cutting sites. HPAF-II cells were transduced with lentivirus packaging a non-targeting control sgRNA (sgNTC #1), sgEP300 #2, or sgEP300 #5 and subsequently selected with puromycin. The genomic DNA was extracted and PCR amplified. The PCR products were purified and subject to Sanger sequencing. In each panel, the reference DNA sequence is shown on the top and the sgRNA sequence is highlighted in yellow. Sanger sequencing signals are shown in the middle and their comparison visualized using TIDE is shown at the bottom.

(D) *EP300* knockout HPAF-II cells were less sensitive to two structurally unrelated PORCN inhibitors. The above mentioned HPAF-II cells (sgNTC #1, sgEP300 #2, and sgEP300 #5) were seeded into 24-well plates at low density and treated with DMSO (0.1% v/v), 100 nM ETC-159, or 10 nM C59 for ~2 weeks. The cell viability was measured by crystal violet staining followed by quantification (n = 3 biological replicates). 3D structures of ETC-159 (PubChem Identifier: CID 86280523; URL: https://pubchem.ncbi.nlm.nih.gov/compound/Etc-159#section=3D-Conformer) and C59 (PubChem Identifier: CID 57519544; URL: https://pubchem.ncbi.nlm.nih.gov/compound/Wnt-C59#section=3D-Conformer) are shown.

(E) A single HPAF-II clone with a homozygous frameshift deletion in *EP300*. A subclone from the HPAF-II sgEP300 #2 cell pool was established. The Sanger sequencing result of the genome edited region is shown with the wildtype reference sequence and sgRNA sequence shown on the top.

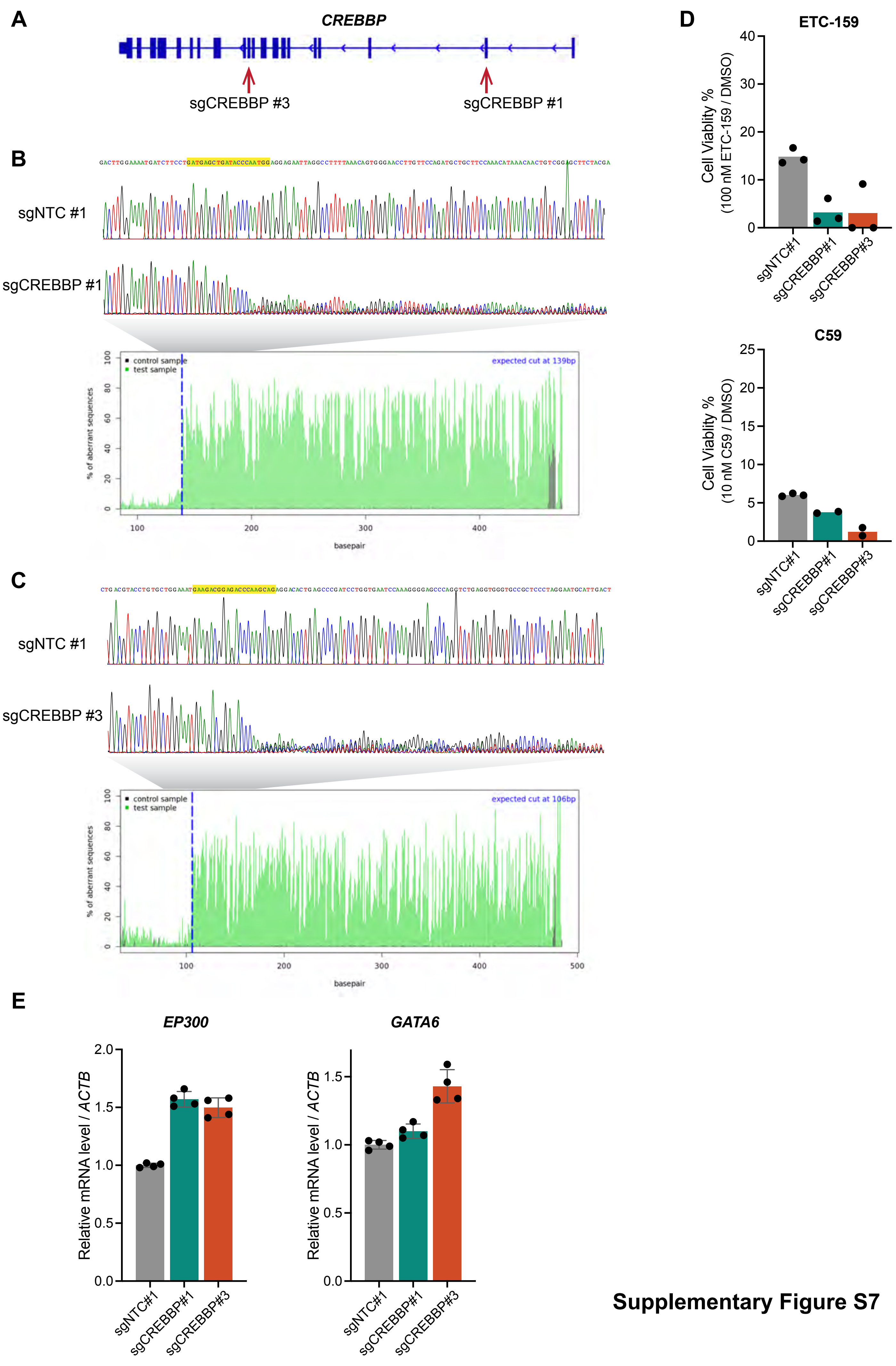

Supplementary Figure S7

Supplementary figure S7. *CREBBP* knockout in HPAF-II cells could not confer resistance to PORCN inhibitors.

(A) Two individual sgRNAs were used to knockout *CREBBP*. The genome positions targeted by these two sgRNAs are labelled in the graph.

(B, C) Both sgRNAs led to considerable indels at the expected cutting sites. HPAF-II cells were transduced with lentivirus packaging a non-targeting control sgRNA (sgNTC #1), sgCREBBP #1, or sgCREBBP #3 and subsequently selected with puromycin. The genomic DNA was extracted and PCR amplified. The PCR products were purified and subject to Sanger sequencing. In each panel, the reference DNA sequence is shown on the top and the sgRNA sequence is highlighted in yellow. Sanger sequencing signals are shown in the middle and their comparison visualized using TIDE is shown at the bottom.

(D) *CREBBP* knockout HPAF-II cells were more sensitive to two structurally unrelated PORCN inhibitors. The above mentioned HPAF-II cells (sgNTC #1, sgCREBBP #1, and sgCREBBP #3) were seeded into 24-well plates at low density and treated with DMSO (0.1% v/v), 100 nM ETC-159, or 10 nM C59 for ~2 weeks. The cell viability was measured by crystal violet staining followed by quantification (n = 2-3 biological replicates).

(E) mRNA levels of *EP300* and *GATA6* in the above mentioned HPAF-II cells were determined by qPCR. Data are represented as mean ± SD (n = 2 biological replicates each with 2 technical replicates). The mRNA level of *EP300* was up-regulated after *CREBBP* knockout, which is a potential compensation mechanism between p300 and CBP. Similarly, the *GATA6* expression level was also slightly higher in the *CREBBP*-knockout cells which could be a response to the up-regulation of p300 expression. These findings correlate well with the above mentioned higher PORCN inhibitor sensitivity of *CREBBP*-knockout cells.

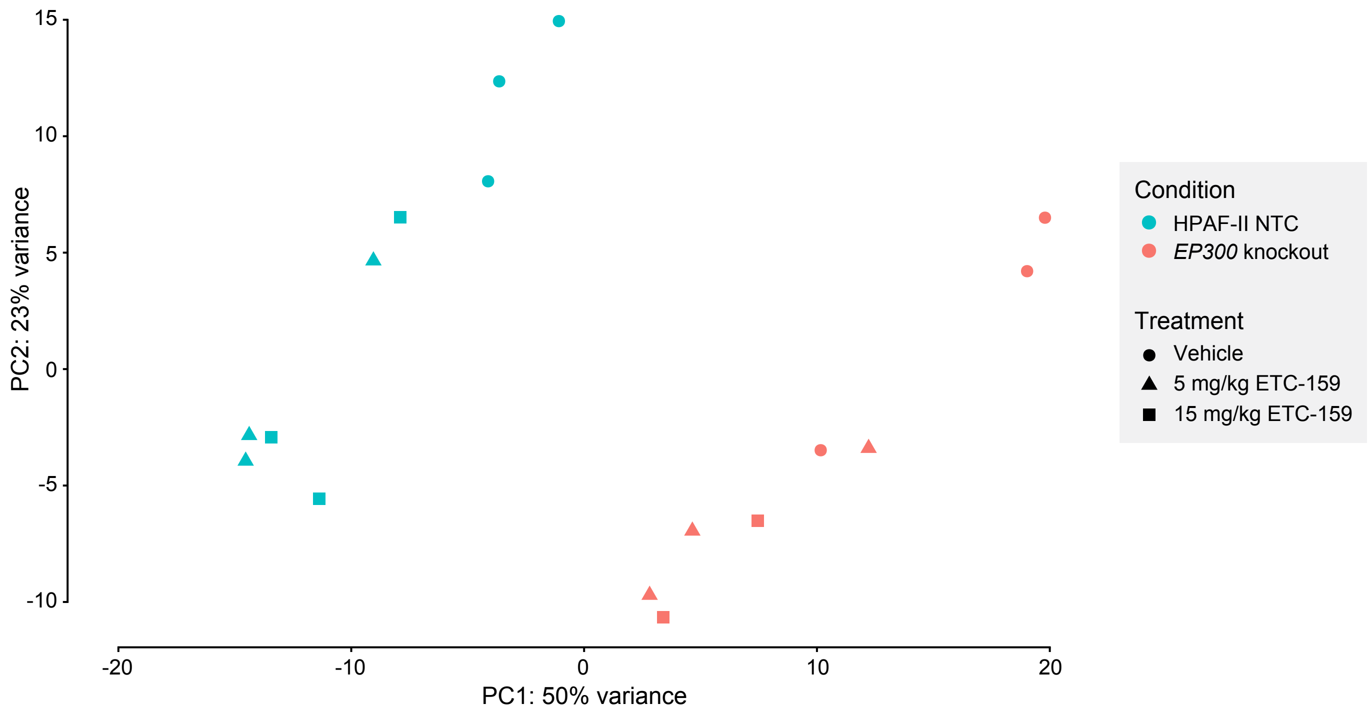

Supplementary Figure S8

Supplementary figure S8. Principal component analysis (PCA) for RNA-seq of *EP300* wildtype or knockout HPAF-II orthotopic xenografts treated with vehicle or ETC-159. Same samples as described in **Fig. 4F**.

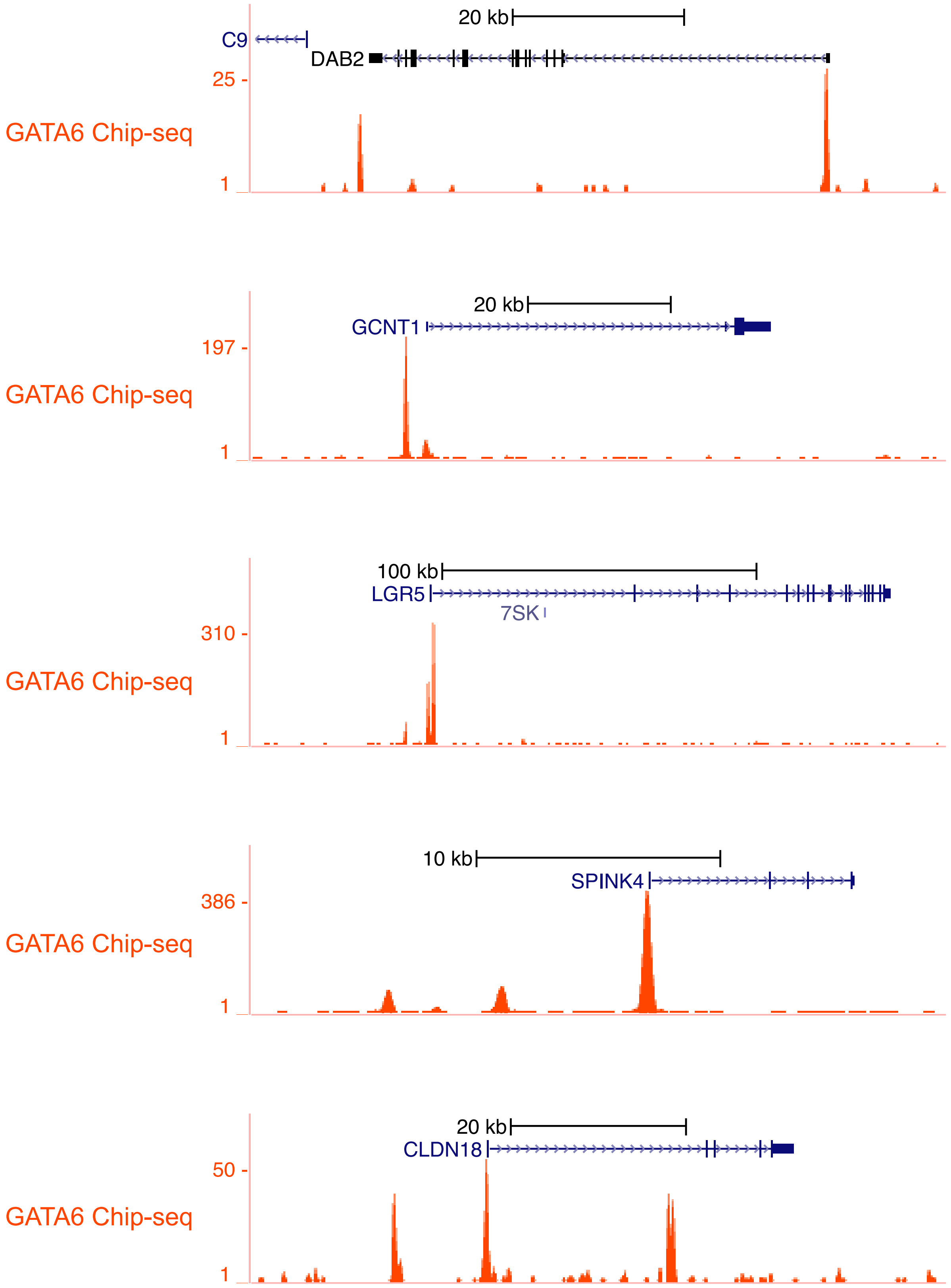

Supplementary Figure S9

Supplementary figure S9. GATA6 binds to the loci of *DAB2*, *GCNT1*, *LGR5*, *SPINK4*, and *CLDN18*.

GATA6 ChIP-seq in Patu8988S cells (from GSE47535) (60) visualized in the UCSC genome browser.

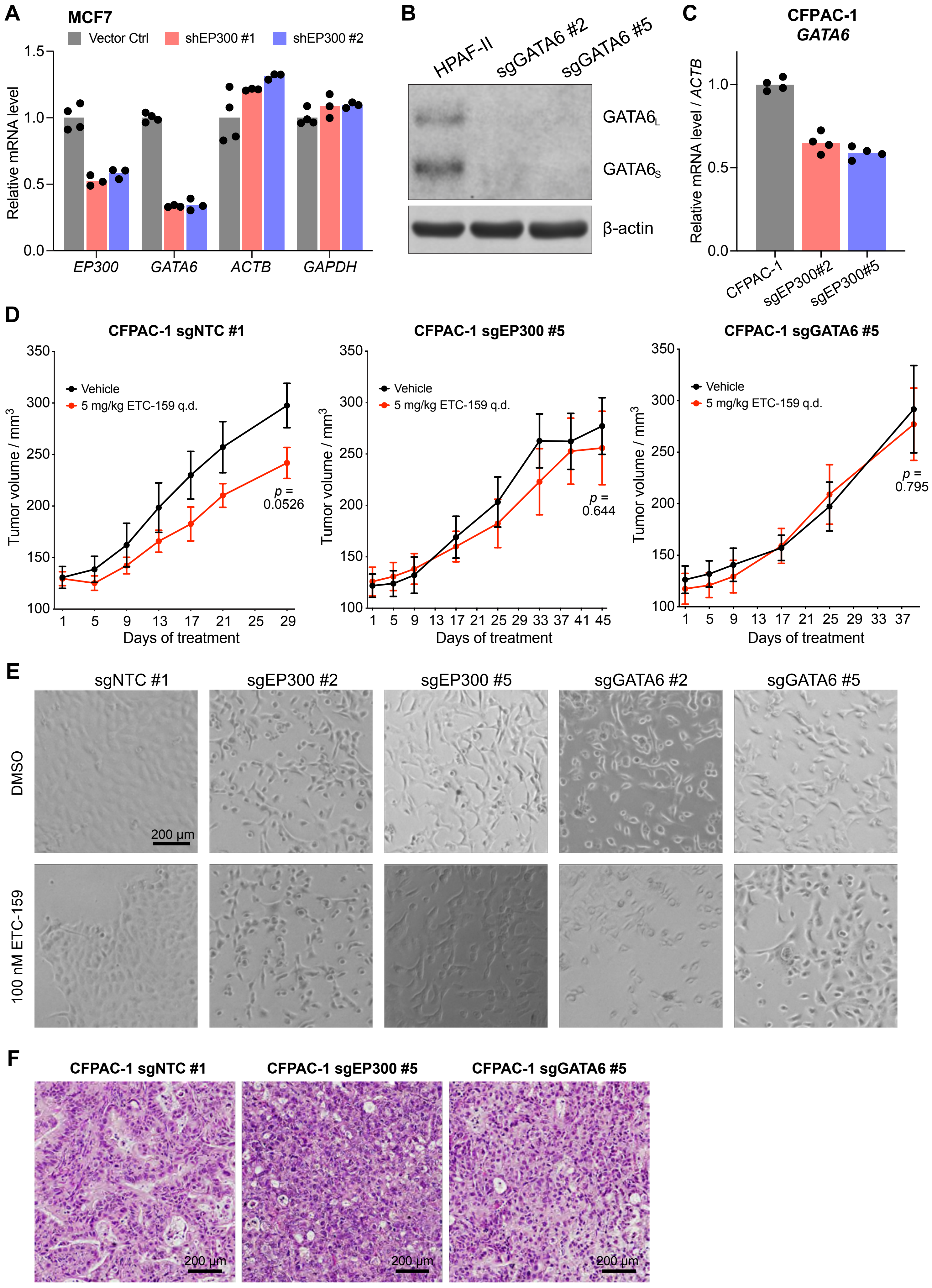

Supplementary Figure S10

Supplementary figure S10. *EP300* knockout conferred resistance to PORCN inhibition and led to pancreatic cancer subtype transition by down-regulating *GATA6* expression.

(A) *EP300* knockdown down-regulated *GATA6* in MCF7 cells (n = 3-4 biological replicates). The expression data were extracted from GSE76200 for *EP300* (probe: 16930534), *GATA6* (probe: 16851383), *ACTB* (probe: 17054823), and *GAPDH* (probe: 16747338).

(B) Two individual sgRNAs were used to knockout *GATA6* in HPAF-II cells. Loss of GATA6 protein was confirmed by Western blot.

(C) *EP300* knockout down-regulated *GATA6* in CFPAC-1 cells. mRNA abundance was determined by RT-qPCR (n = 2 biological replicates each with 2 technical replicates).

(D) *EP300* knockout and *GATA6* knockout conferred resistance to ETC-159 in CFPAC-1 tumors. CFPAC-1 cells transduced with a non-targeting control sgRNA (sgNTC #1), sgEP300 #5, or sgGATA6 #5 were subcutaneously injected into NSG mice. After tumor establishment, tumor-bearing mice from each group were randomized into two arms (n = 8 tumors per arm) and treated with vehicle or 5 mg/kg ETC-159 q.d., respectively. Data are represented as mean ± SEM. *p* values of two-tailed unpaired *t*-test for tumor volumes of the last time point between vehicle and ETC-159 arms are shown in the graphs.

(E, F) *EP300* knockout and *GATA6* knockout changed the morphology of CFPAC-1 cells *in vitro* and *in vivo*. In 2D culture, CFPAC-1 sgNTC #1 cells grew as monolayer clusters. *EP300* knockout and *GATA6* knockout cells formed clusters when they reached confluency but still grew as spindle-shaped mesenchymal-like cells in the leading edge. This was not affected by ETC-159 treatment. Representative brightfield images are shown (n = 3 biological replicates) **(E)**. Vehicle-treated tumors from **D** were subject to H&E staining **(F)**.

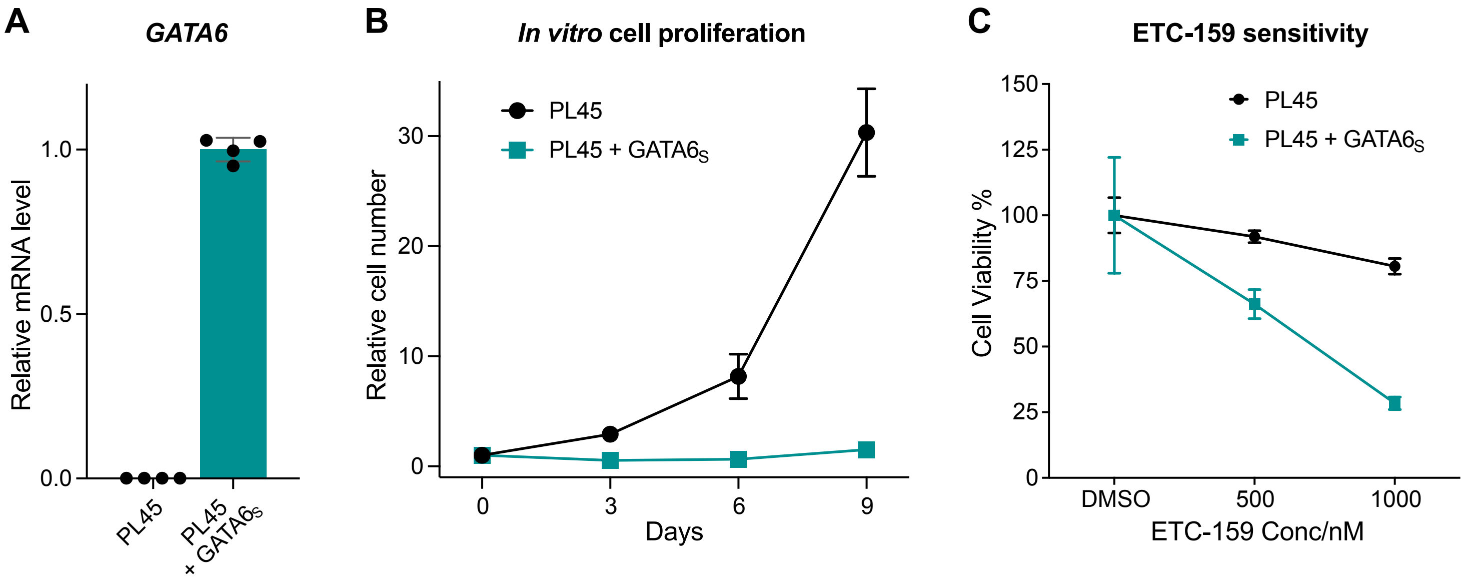

Supplementary Figure S11

Supplementary figure S11. Stably expression of $GATA6_S$ in PL45 cells (*RNF43*/*EP300*-mutant) slowed down cell proliferation and increased PORCN inhibitor sensitivity.

(A) *GATA6* mRNA abundance was determined by RT-qPCR (n = 2 biological replicates each with 2 technical replicates). Data are represented as mean ± SD.

(B) Equal number of PL45 or PL45 + $GATA6_S$ cells were seeded in 6-well plates at day 0 and harvested and counted at the indicated time points. Data are represented as mean ± SD (n = 3 biological replicates).

(C) Cells were seeded in 24-well plates at low density and treated with DMSO (0.1% v/v) or the indicated concentration of ETC-159. The cell viability was measured by crystal violet staining followed by quantification. Data are represented as mean ± SEM (n = 3 biological replicates).

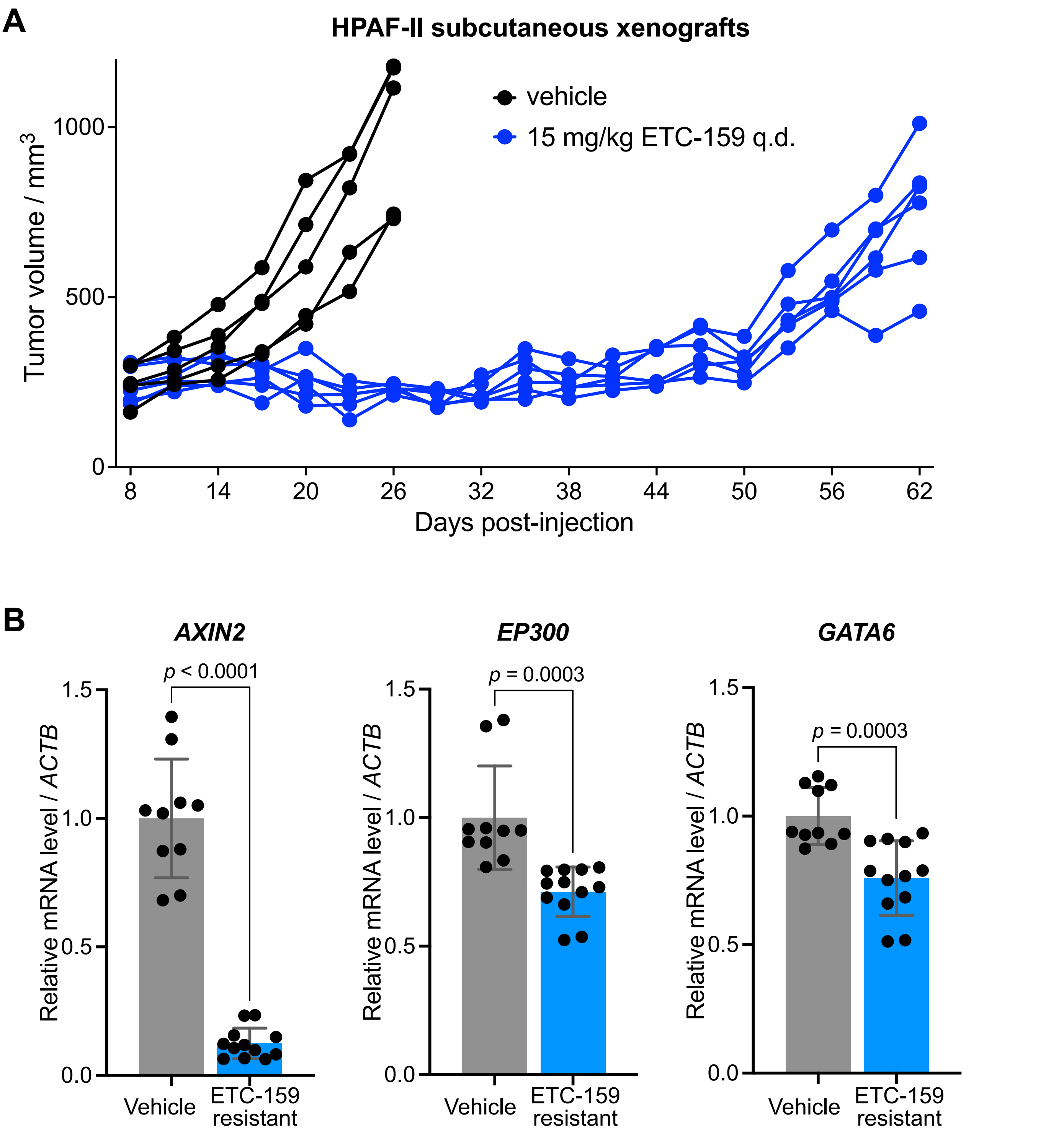

Supplementary Figure S12

Supplementary figure S12. mRNA levels of *EP300* and *GATA6* were down-regulated in HPAF-II tumors that acquired resistance to ETC-159.

(A) HPAF-II cells were subcutaneously implanted in NSG mice. After tumor establishment (8 days post-injection), these mice were treated with 15 mg/kg ETC-159 once daily until confirmed drug resistance emerged. Note that daily ETC-159 treatment continued to the end of the study (day 62). Growth curves of individual tumors are shown.

(B) Relative mRNA abundance of *AXIN2*, *EP300*, and *GATA6* in vehicle-treated HPAF-II tumors (harvested on day 26) and ETC-159-treated tumors (harvested on day 62) six hours after the last gavage dose. Tumors from five vehicle-treated tumors and six ETC-159-treated tumors were used for qPCR analysis, with technical replicates for each sample. Data are represented as mean ± SD. *p* values of unpaired t test are shown. *AXIN2* is still suppressed in the ETC-159-treated tumors, indicating ETC-159 still has on-target effects.

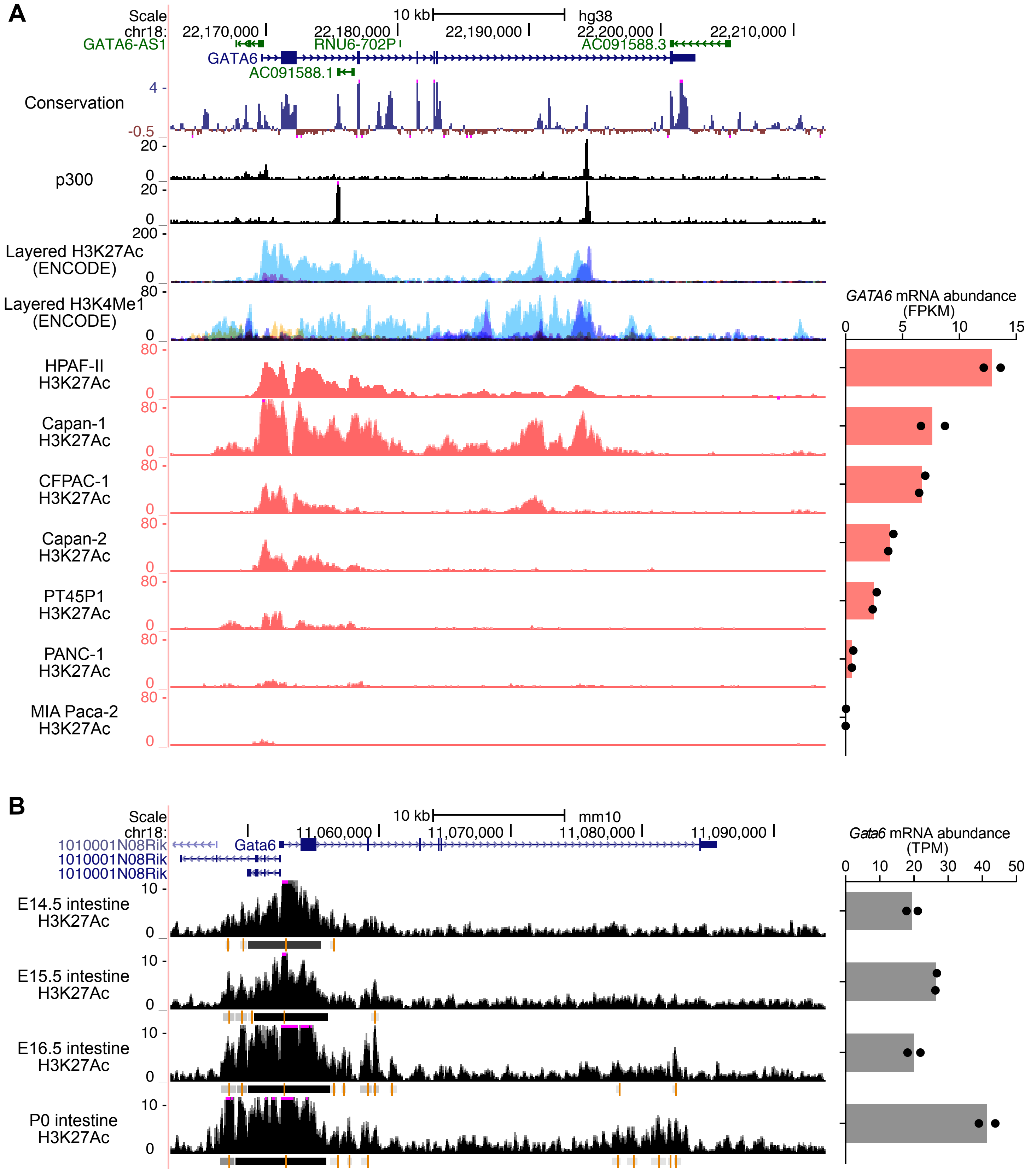

Supplementary Figure S13

Supplementary figure S13. H3K27Ac signals in the *GATA6* promoter and enhancer regions correlate with *GATA6* expression levels.

(A) The UCSC conservation track (100 vertebrate species)(shown in **Fig. 5H**), ENCODE p300 ChIP-seq track (upper: K562 cells, ENCSR000EGE; lower: HepG2 cells, ENCSR000BLW) (shown in **Fig. 5H**), ENCODE layered H3K27Ac ChIP-seq track (7 cell lines), ENCODE layered H3K4Me1 ChIP-seq track (7 cell lines), and H3K27Ac ChIP-seq tracks for a panel of pancreatic cancer cell lines (GSE64557) (105) in the *GATA6* locus (HPAF-II track shown in **Fig. 5H**). The *GATA6* mRNA abundances (FPKM derived from GSE64558 (105)) in the pancreatic cancer cell lines are shown next to the corresponding H3K27Ac tracks.

(B) ENCODE H3K27Ac ChIP-seq tracks for mouse intestines collected at the indicated time points. Statistically significant peaks are marked with lines and rectangles under each track. The *Gata6* mRNA abundances (TPM derived from the corresponding publication (106)) in mouse intestines collected at the indicated time points are shown next to the corresponding H3K27Ac tracks.

A

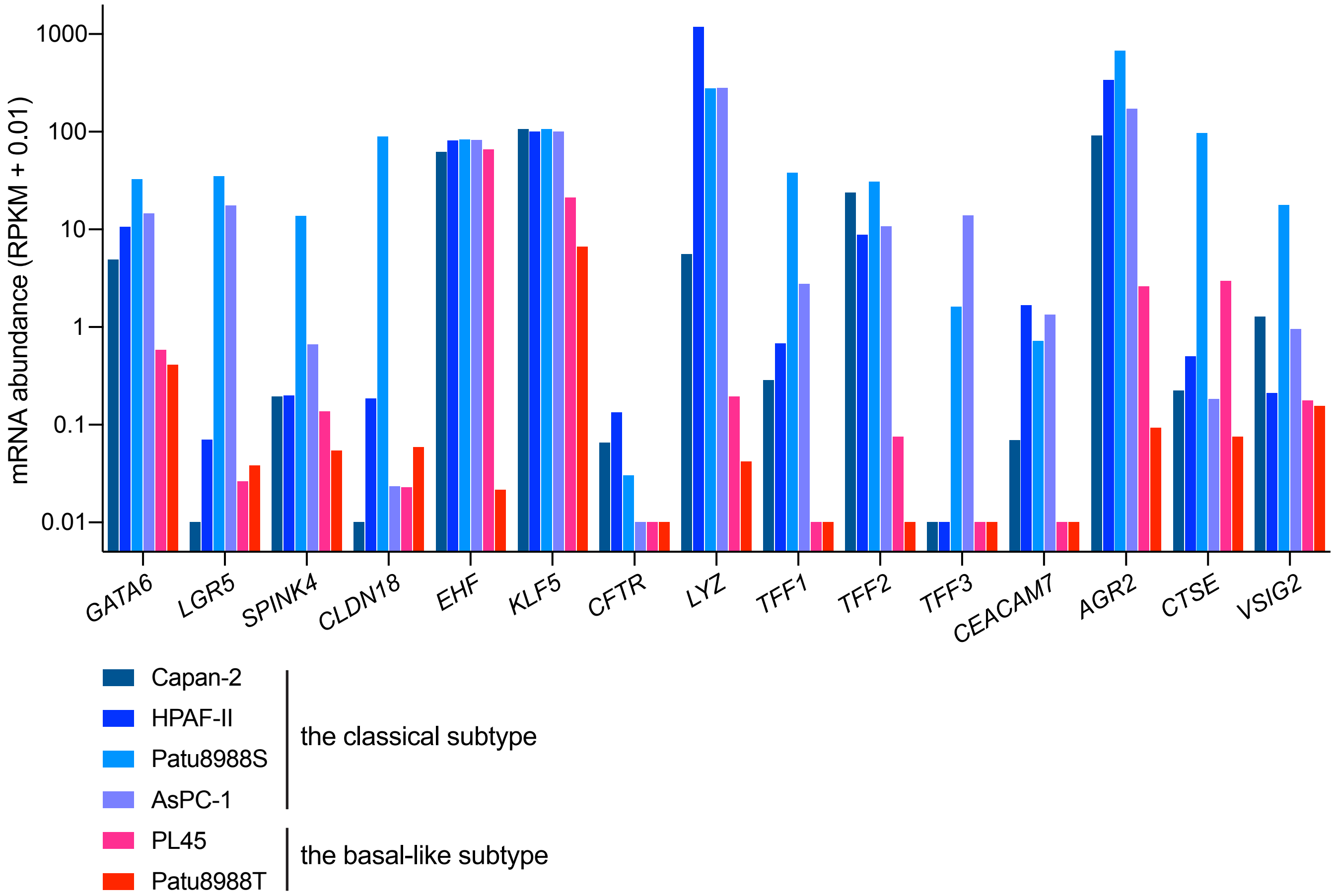

B

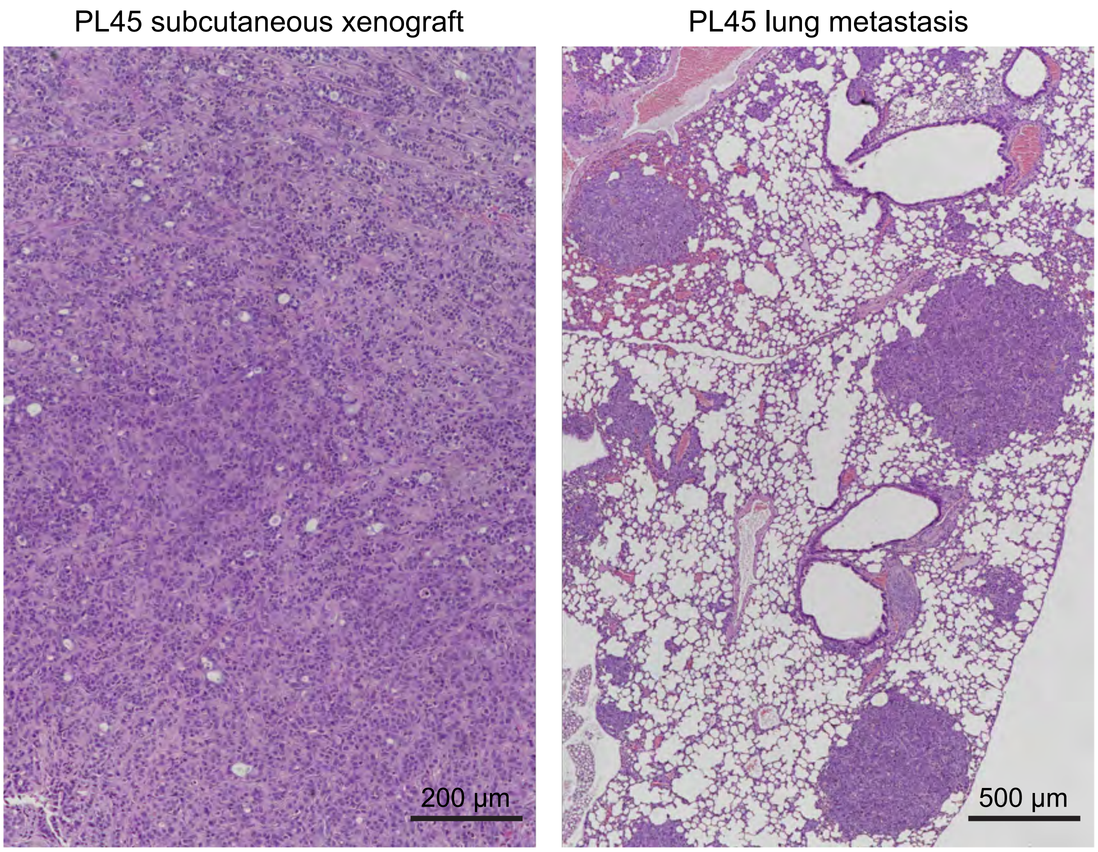

Supplementary Figure S14

Supplementary figure S14. ***RNF43/EP300*-mutant pancreatic cancer cell lines show basal-like subtype features.**

(A) mRNA abundances of *GATA6*, GATA6 target genes, pancreatic lineage, differentiation-related, epithelial, and/or classical subtype markers in a panel of *RNF43*-mutant pancreatic cancer cell lines. The RPKM values were extracted from (45).

(B) Representative images of H&E staining of PL45 subcutaneous xenografts and lung metastasis.

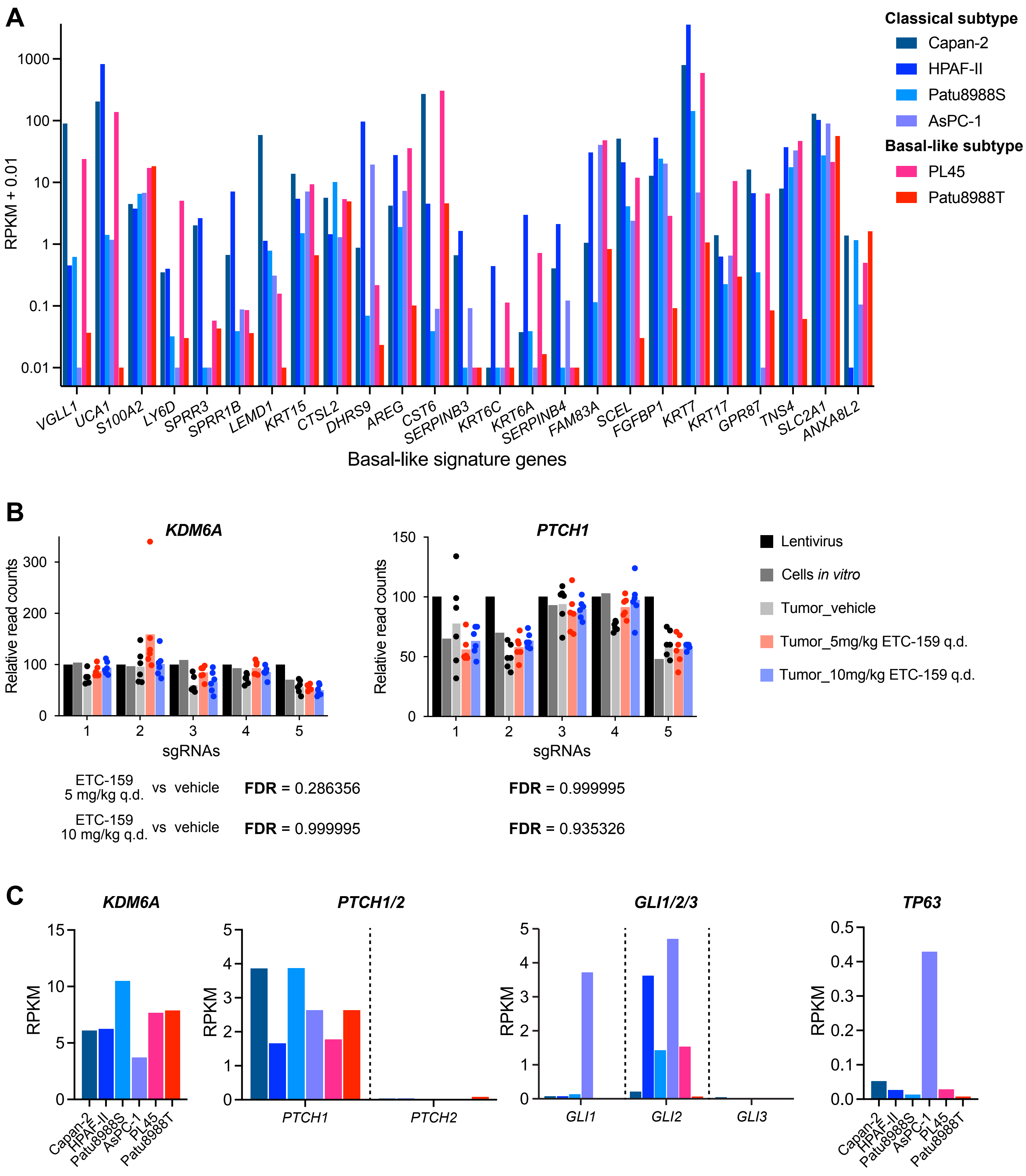

Supplementary Figure S15

Supplementary figure S15. (A) Basal-like gene signature is not up-regulated in the *RNF43*/*EP300*-mutant *GATA6*-low pancreatic cancer cell lines, compared to the *RNF43*-mutant *EP300*-wildtype *GATA6*-high pancreatic cancer cell lines. mRNA levels of a panel of basal-like subtype signature genes (genes highly expressed in basal-like subtype pancreatic tumors, derived from (65)) are shown. The RPKM values were extracted from (45).

(B) Individual sgRNA read count change in all the samples for *KDM6A* and *PTCH1* from our CRISPR screens.

(C) mRNA levels of *KDM6A*, *PTCH1*/*2*, *GLI1*/*2*/*3*, and *TP63* in the same panel of cell lines shown in **(A)**. *PTCH1* is the dominantly expressed *PTCH* in pancreatic cancer whereas the expression of its paralog *PTCH2* is barely detected in HPAF-II cells. *GLI2* is the dominantly expressed *GLI* compared to *GLI1*/*3* in HPAF-II cells. The RPKM values were extracted from (45).

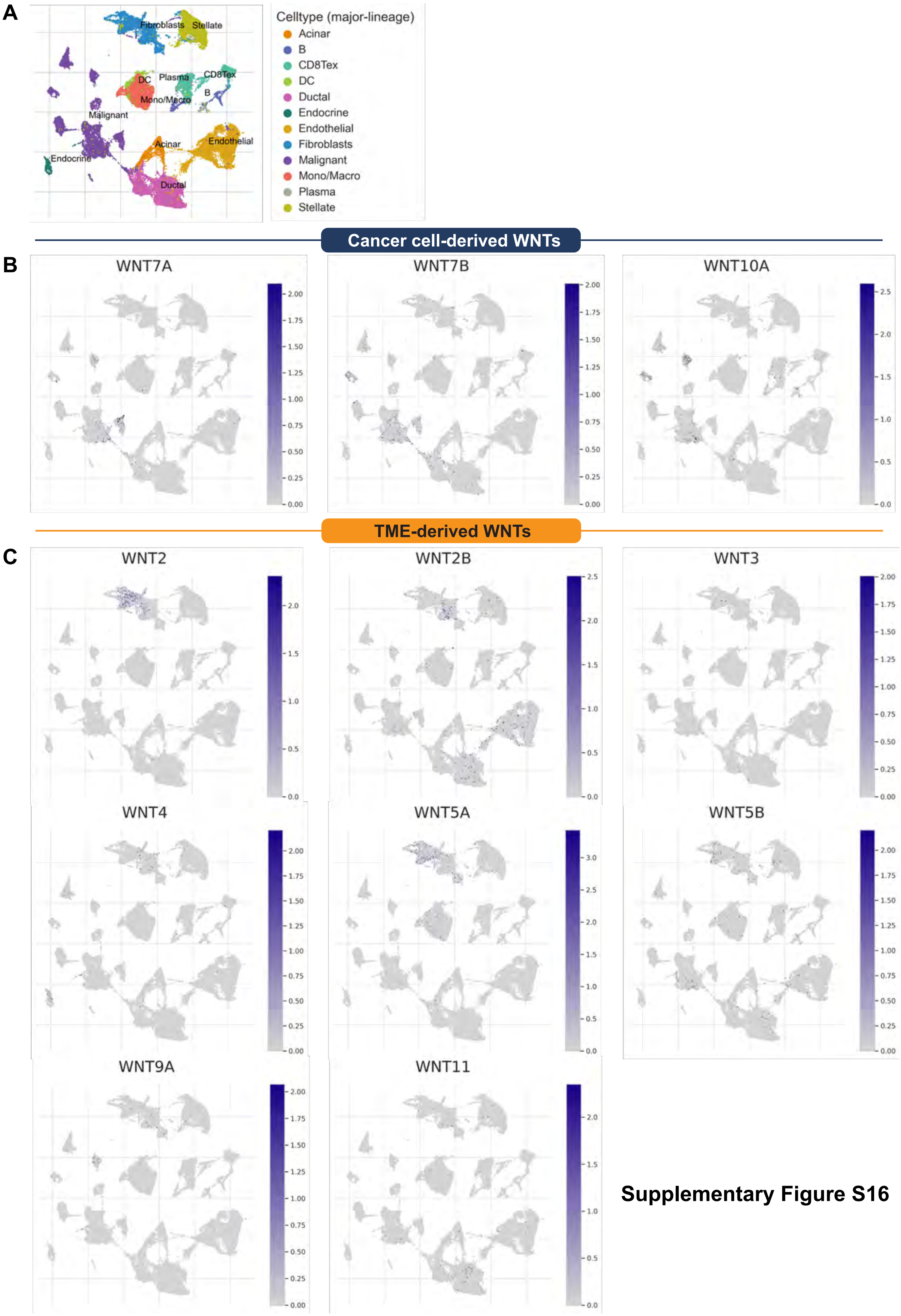

Supplementary Figure S16

Supplementary figure S16. Diverse origins of Wnt ligands in pancreatic tumors.

Single-cell RNA-seq results of pancreatic tumors (73) visualized with TISCH website.

(A) The types of cells in primary pancreatic tumors.

(B, C) Robustly detectable Wnt ligands expressed in pancreatic tumors come from cancer cells **(B)** or tumor microenvironment (TME) **(C)**.

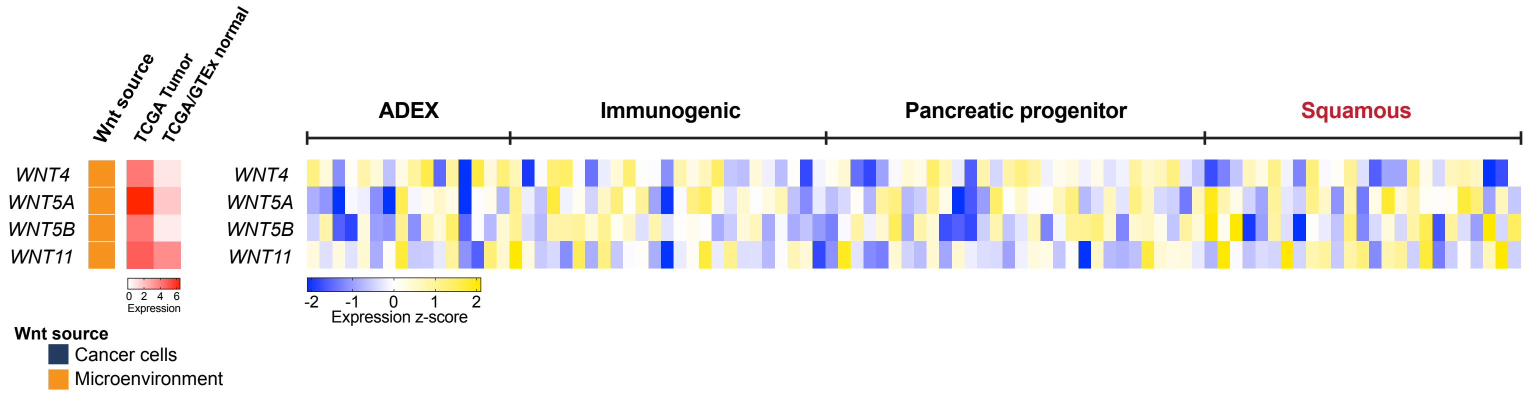

Supplementary Figure S17

Supplementary figure S17. ***WNT4*****,** ***WNT5A*****,** ***WNT5B*****, and** ***WNT11*** **had similar expression levels in different subtypes of pancreatic cancer.**

Gene expression data were analyzed and represented as in Fig. 8A.

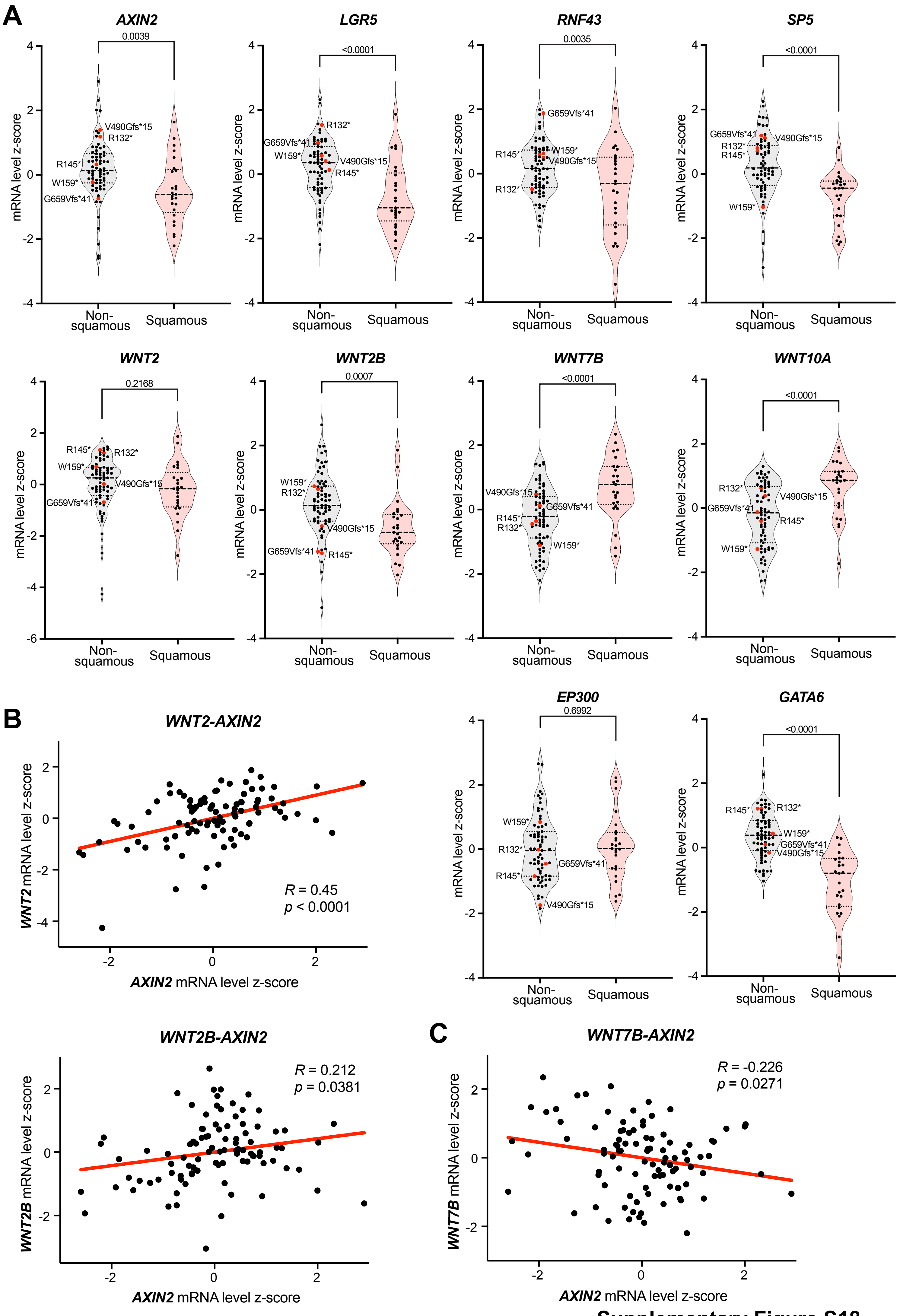

Supplementary Figure S18

Supplementary figure S18. (A) mRNA levels of Wnt target genes (*AXIN2*, *LGR5*, *RNF43*, *SP5*), stromal Wnts (*WNT2* and *WNT2B*) and epithelial Wnts (*WNT7B* and *WNT10A*), *EP300*, and *GATA6* in the Bailey et al PDAC cohort (corresponding to **Fig. 8A**). Samples are classified into the squamous and non-squamous (ADEX, immunogenic, and pancreatic progenitor subtypes) subclasses. Each dot represents one sample and the *RNF43*-mutant samples are shown as red dots with the corresponding *RNF43* mutation labelled nearby. *p* values of two-tailed unpaired *t*-test are shown.

(B) Stromal Wnts are more likely to be driving Wnt/β-catenin signaling. The mRNA levels of stromal *WNT2* and *WNT2B* positively correlate with *AXIN2* expression whereas **(C)** the epithelial *WNT7B* shows a negative correlation (n = 96). *R* and *p* values of simple linear regression are shown.
